# Supplementary material for: Splice-Modulating Oligonucleotide QR-110 Restores CEP290 mRNA and Function in Human c.2991+1655A>G LCA10 Models
Source: Mol Ther Nucleic Acids. 2018 Jul 23;12:730–40. doi: 10.1016/j.omtn.2018.07.010 (PMC6092551; doi:10.1016/j.omtn.2018.07.010)
Supplement: Document S1. Supplemental Materials and Methods, Figures S1–S7, and Tables S1–S8 [file mmc1.pdf]

OMTN, Volume *12*

Supplemental Information

Splice-Modulating Oligonucleotide QR-110 Restores *CEP290* mRNA and Function in Human c.2991+1655A>G LCA10 Models

Kalyan Dulla, Monica Aguila, Amelia Lane, Katarina Jovanovic, David A. Parfitt, Iris Schulkens, Hee Lam Chan, Iris Schmidt, Wouter Beumer, Lars Vorthoren, Rob W.J. Collin, Alejandro Garanto, Lonneke Duijkers, Anna Brugulat-Panes, Ma'ayan Semo, Anthony A. Vugler, Patricia Biasutto, Peter Adamson, and Michael E. Cheetham

Supplemental Information

Supplementary Figures

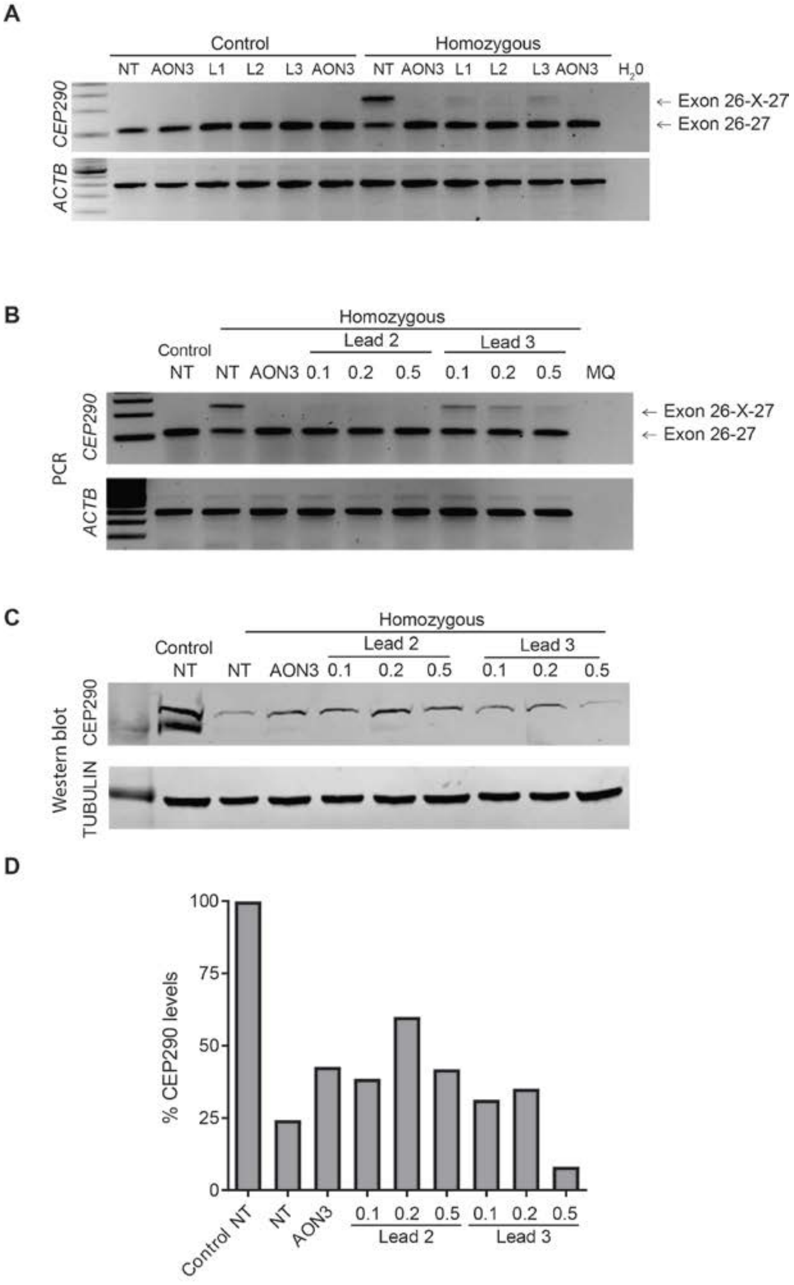

Figure S1. Screening of splice modulating oligonucleotides. (A) Control and c.2991+1655A>G homozygous fibroblasts were transfected with 100 nM each of Lead 1, 2 (i.e. QR-110), 3 or AON3 (Collin et al 2012) oligonucleotide, using Fugene reagent and analyzed for *CEP290* by RT-PCR. Oligo concentrations used here are higher than those of experiments described in the main text due to the use of different transfection reagent. **(B)** Homozygous fibroblasts were treated with 3 concentrations (0.1, 0.2, 0.5 µM) of Lead 2 (i.e. QR-110) or Lead 3 and one concentration (100 nM) of control oligo using Fugene reagent and *CEP290* transcripts were analyzed by RT-PCR and (**C**) CEP290 levels by Western blot. **(D)** Protein signal was quantified by densitometry analysis, normalized by tubulin. NT not treated. n=1. At highest oligo concentration, a reduction in the CEP290 levels was noticed, probably due to cytotoxicity.

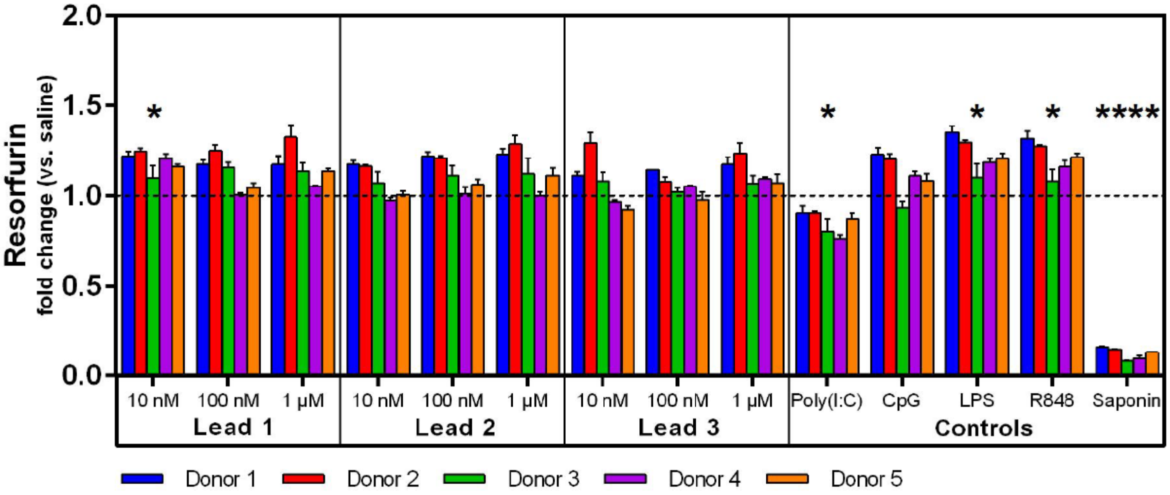

Figure S2. Viability assay of human PBMC treated with lead oligos. Relative number of viable PBMC expressed as fold change of Resorufin fluorescence compared to saline treated PBMC after 24 hours exposure to Lead 1 to 3 (10nM, 100nM or 1µM) or the positive controls Poly(I:C) (1µg/ml), CpG (10µg/ml), LPS (100ng/ml) and R848 (1µM). For all individual biological replicates, fold changes were calculated by normalizing measured RFU against geometric mean of corresponding triplicate saline control. Results are shown per individual donor as the mean+SEM of the triplicate fold change, normalized against the mean of its corresponding saline control (dotted line). Repeated measures One-way ANOVA with Dunnett test for multiple corrections (compared to saline) was performed on the fold change values. Statistical significance is depicted as *(p>0.05) or **** (p>0.0001).

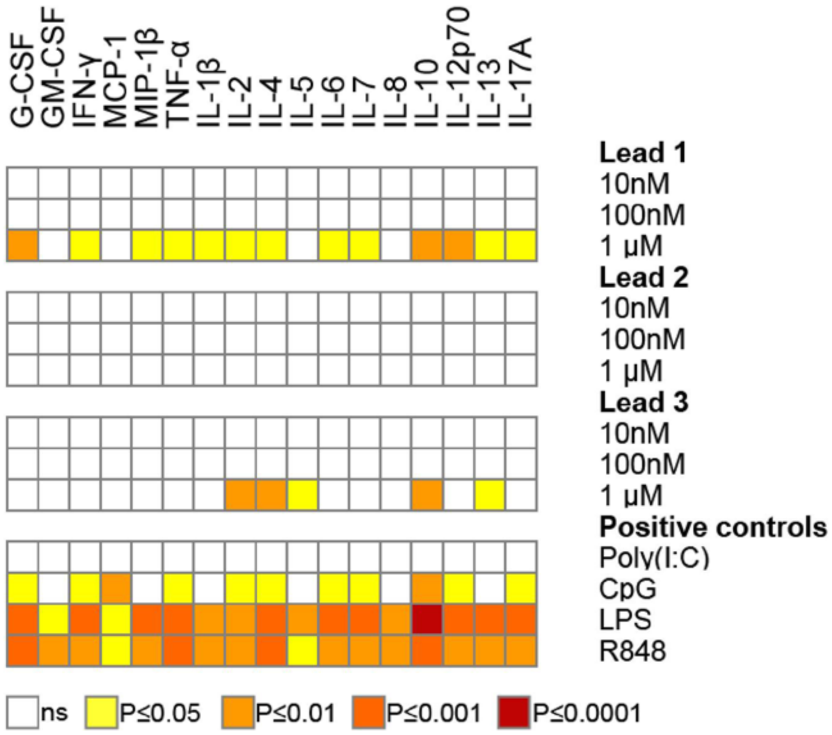

Figure S3. Cytokine production by human PMBC treated with lead oligos. Heat map depicting the statistical significance of cytokine changes compared to saline-treated PBMC, in culture supernatant 24 hours after stimulation of PBMC with Lead 1 to 3 (10nM, 100nM or 1µM) or the positive controls Poly(I:C) (1µg/ml), CpG (10µg/ml), LPS (100ng/ml) and R848 (1µM). Geometric mean of the five human donors with triplicate measurements each. P-values were determined using the Friedman test with Dunn's post-hoc test. Not-significant: ns.

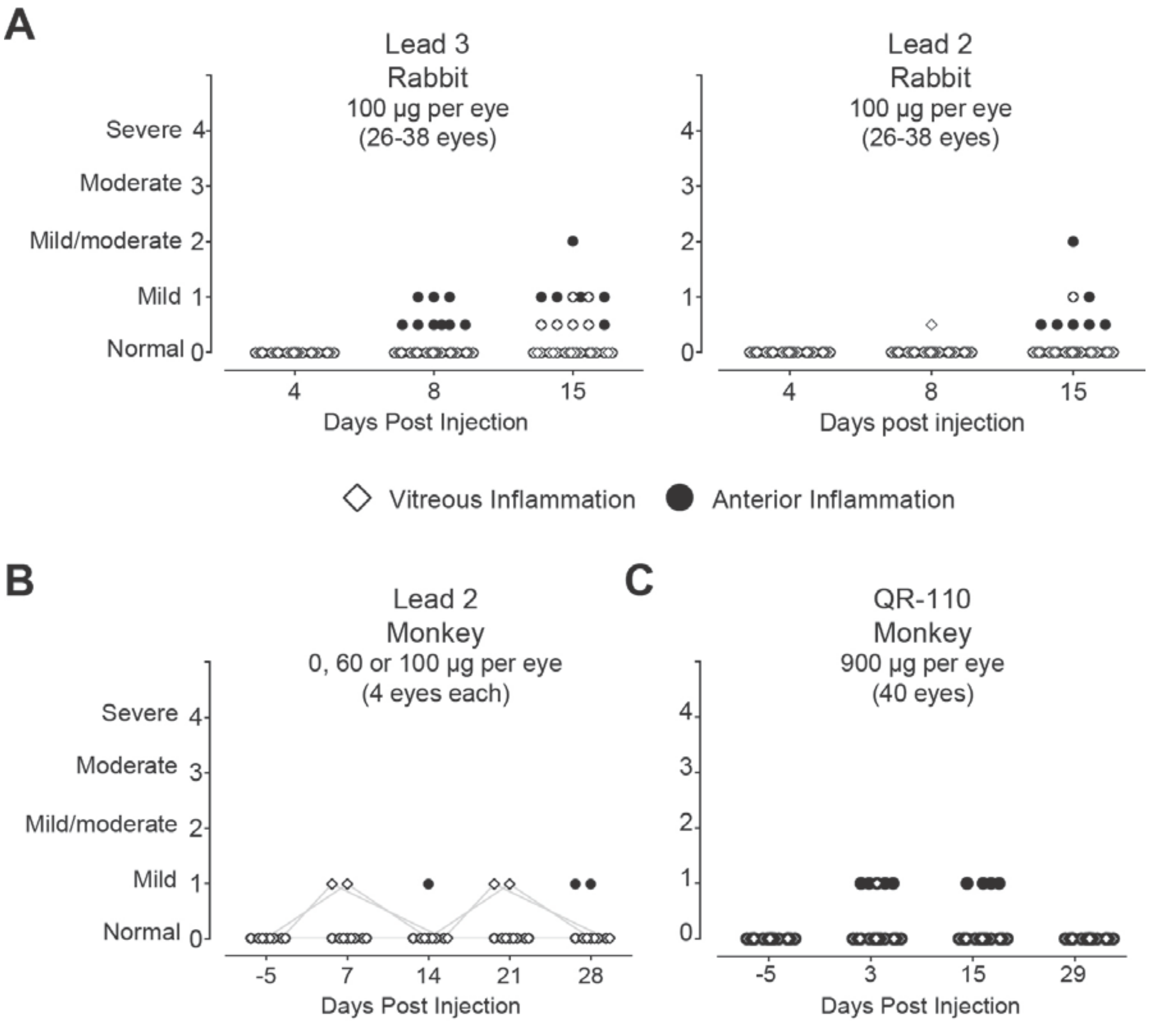

Figure S4. In-vivo tolerability studies. Animals received a single intravitreal injection of test article or just the PBS vehicle. Ophthalmic examinations were performed by a veterinary ophthalmologist using slitlamp biomicroscope and indirect/direct ophthalmoscopy and inflammation, if any, was scored in anterior and posterior segments. (**A**) Dutch-Belted rabbits received a single intravitreal injection of 100 µg (14 µM) per eye of either Lead 2 or Lead 3 oligonucleotide and were assessed on day 4, 8 and 15. Six animals each were sacrificed at each assessment time point for further analysis thus different number of eyes (38-26) were assessed at each time point. (**B**) Cynomolgus monkeys received a single intravitreal injection of either 0, 60 (6 µM) or 100 µg (10 µM) per eye (2 animals per dose level) and the inflammation was assessed. The findings from the animals treated with either 60 or 100 µg are plotted together. Findings from the control animals are connected by the grey lines which highlight the absence of any trend in inflammation in oligo treated animals and only minor fluctuations were seen. (**C**) In a separate study, cynomolgus monkeys were dosed with 900 (93 µM) µg QR-110 per eye and were assessed at 5 days pre-dosing and 3, 15 and 29 days post-posting. *In vivo* doses and the corresponding concentration in the vitreous humor are shown in Table S7.

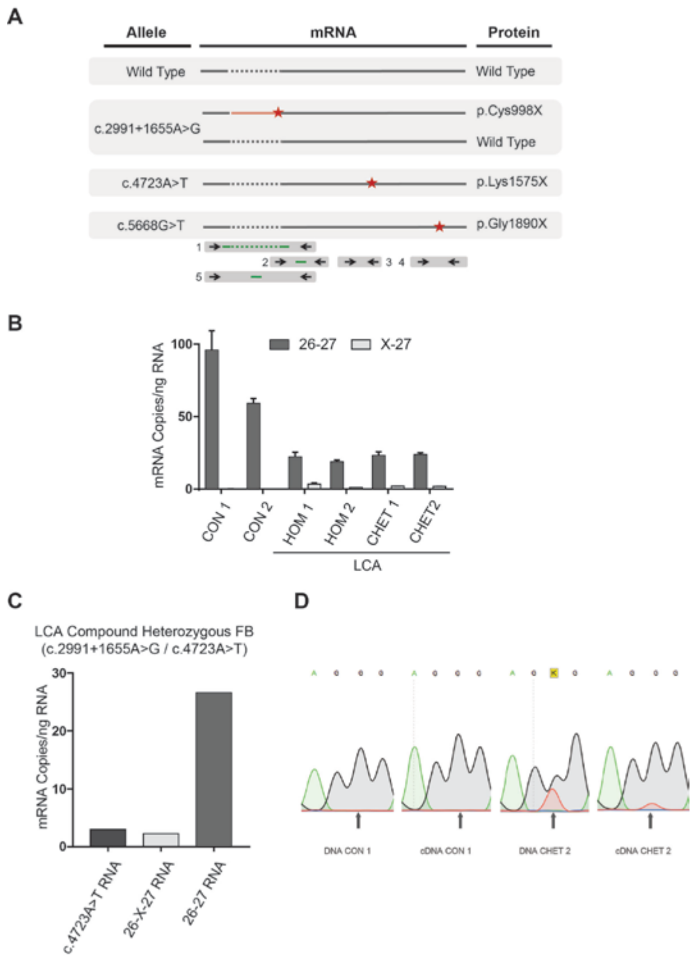

Figure S5. *CEP290* levels are reduced in c.2991+1655A>G homozygous and compound heterozygous LCA patient fibroblasts and the mutant *CEP290* transcripts are degraded by NMD. (**A**) Schematic representation of different *CEP290* alleles present in the LCA models investigated in this study and the corresponding mRNA and protein produced. All alleles produce one type of mRNA apart from the c.2991+1655A>G which produces both wild-type and mutant transcripts. PCR assay design strategy to specifically detect different transcripts is shown at the bottom of the schematic. Wild-type PCR assay (1) relies on the junction of exon 26-27, so this assay also detects transcripts containing c.4723A>T and c.5668G>T. The c.2991+1655A>G PCR assays X-27 (2) and 26-X-27 (5), and c.4723A>T PCR assay (3) are specific for the mutations and do not detect any other transcript. The c.5668G>T assay (4) amplifies the region around the c.5668G>T mutation and sequencing was used to specifically detect the c.5668G>T isoform. The position of stop codons is shown by a red star, PCR primers by arrows and ddPCR probe positions are shown in green**.** (**B**) Wild-type and c.2991+1655A>G *CEP290* expression is measured using ddPCR in two healthy control (CON); and two homozygous (HOM) and two compound heterozygous (CHET) fibroblast cell lines isolated from LCA patients. Control cells expressed wild-type *CEP290* exclusively. In LCA cells c.2991+1655A>G transcripts were only 10% of the wild-type transcripts levels, but wild-type transcripts levels themselves were 23%-36% of the healthy control cells. (**C**) Three different *CEP290* transcripts expressed in the compound heterozygous cell line 1 (c.2991+1655A>G / c.4723A>T) were quantified using isoform specific ddPCR assays. High wild-type levels with significantly low c.4723A>T levels comparable to c.2991+1655A>G transcripts point to NMD mediated degradation of transcripts with the premature stop codon. (**D**) PCR primers binding to exon 41 were used to amplify the region around the c.5668G>T mutation from DNA and cDNA of control (CON) and compound heterozygous (c.2991+1655A>G / c.5668G>T) fibroblasts and sequenced using Sanger sequencing. No c.5668G>T signal was detected in control cells (highlighted with an arrow). In compound heterozygous cells, as expected the c.5668G>T mutation was ~50% of the DNA, whereas the levels were much lower in cDNA, indicating NMD of the c.5668G>T transcript.

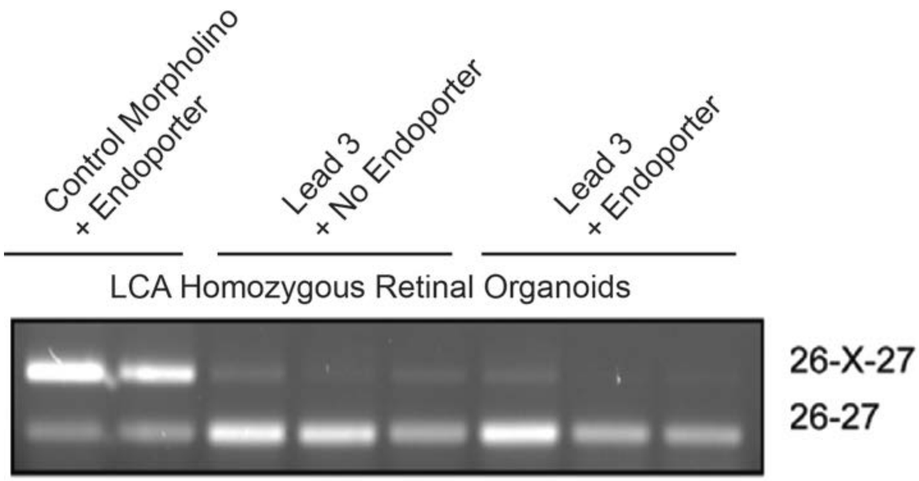

Figure S6. Lead 3, a *CEP290*-directed 2' O methyl phosphorothioate oligo, demonstrates efficacy with and without transfection reagent. Homozygous LCA10 retinal organoids were treated with culture medium containing 10 μM control morpholino (Parfitt et al 2016) with Endoporter, Lead 3 with Endoporter or Lead 3 gymnotically twice per week, starting from day 96 of maturation, for 4 weeks and *CEP290* transcripts were analyzed by RT-PCR.

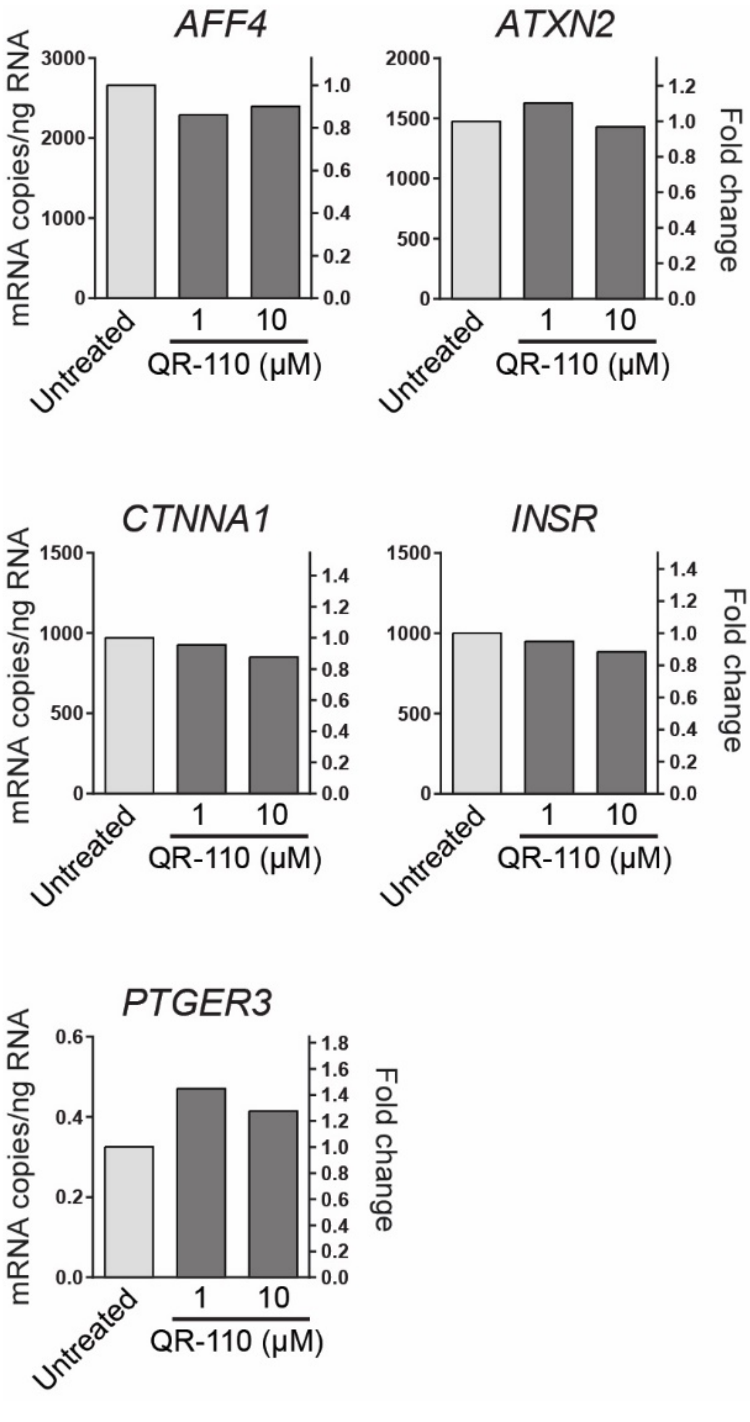

Figure S7. QR-110 effect on a set of potential off-targets investigated in homozygous LCA10 retinal organoids. Transcript levels were determined by gene-specific one-step RT-ddPCR. Results were normalized using housekeeping genes (*GUSB*, *HPRT*) and as expressed as copies per ng of RNA input. All values were expressed as mean ± SEM (one retinal organoid per treatment condition).

Tables

Supplementary Table 1. Cell lines

**Name**	**Genotype**	**Description**
CON1	Wild-type	Control fibroblasts
CON2	Wild-type	Control fibroblasts
HOM1	c.2991+1655A>G / c.2991+1655A>G	p.Cys998X homozygous fibroblasts
HOM2	c.2991+1655A>G / c.2991+1655A>G	p.Cys998X homozygous fibroblasts
CHET1	c.2991+1655A>G / c.4723A>T	p.Cys998X/p.Lys1575X compound heterozygous fibroblasts
CHET2	c.2991+1655A>G / c.5668G>T	p.Cys998X/p.Gly1890X compound heterozygous fibroblasts
HOM3 RO	c.2991+1655A>G / c.2991+1655A>G	p.Cys998X homozygous retinal organoids

Supplementary Table 2. Details of the oligonucleotides used.

**Name**	**Sequence (5' → 3')**	**Manufacturer**	**Chemistry**
QR-110	GGUGGAUCACGAGUUCA	Biospring	Phosphorothioated backbone and 2' O-methyl RNA bases
Lead 1	GGCUCACAUCUGUAAUC	Axolabs GmbH	
Lead 2 (QR-110)	GGUGGAUCACGAGUUCA	Axolabs GmbH	
Lead 3	GGAUAGGUAUGAGAUAC	Axolabs GmbH	
Scrambled	AUAGUAACGGAUUGAGG	IDT DNA	

Supplementary Table 3. Different media used for organoid differentiation.

**Name**	**Contents**
Fibroblast culture medium	DMEM – high glucose AQmedia (Sigma-Aldrich), 20% fetal bovine serum (Biowest),1% sodium pyruvate (Sigma-Aldrich)
Embryoid body (EB) medium	Glasgow's MEM (GMEM, Life Technologies), 20% KOSR (Life Technologies), 1mM NEAA, 1mM sodium pyruvate, 100µM β-mercaptoethanol
EB1 medium	Glasgow's MEM (GMEM, Life Technologies), 20% KnockOut Serum Replacement (KOSR, Life Technologies), 1mM NEAA, 1mM sodium pyruvate, 100µM β-mercaptoethanol, 10µM Y-27632, 3µM IWR1e (Calbiochem), 2% Matrigel (Corning)
EB2 medium	GMEM, 20% KOSR, 1mM NEAA, 1mM sodium pyruvate, 100µM β-mercaptoethanol, 10% FBS, 1% Matrigel, 10µM Y-27632, 100nM smoothened agonist (SAG; Enzo Life Sciences)
Neural retinal differentiation (NR) medium	DMEM-F12, 10% FBS, 1% N2 supplement (Life Technologies), 0.5µM retinoic acid (RA; Tocris)

Supplementary Table 4. Primers and probes used for RT-PCR and ddPCR.

RT-PCR assays		
**Gene name**	**Forward primer**	**Reverse primer**
CEP290	TGCTAAGTACAGGGACATCTTGC	AGACTCCACTTGTTCTTTTAAGGAG
CRX	CCCACTATTCTGTCAACGCCT	TCTTGGCAAACAGTGCCTCC
NRL	CACTGACCACATCCTCTCGG	GAGGGTTCCCGCTTTACCTC
NR2E3	TGGTCCTCTTCAAGCCAGAGA	TTTCACCTCCACCCCCACTA
GAPDH	TGCACCACCAACTGCTTAG	GGATGCAGGGATGATGTTC
c.5668G>T *CEP290* (4)	GGCAAACCCCTGACAGATAA	GGTTTTAGGTCTACTTCCTC

Custom-designed ddPCR assays			
Gene name (Fig. S5A)	Forward primer	Reverse primer	Probe
Wild-Type *CEP290* (1)	GCTAAGTACAGG GACATCTTGC	AGACTCCACTTGTTCTTT TAAGGAG	/56-FAM/ CTGGAGT+GT+GA+A+AA+C A+TC -IABkFQ
c.2991+1655A>G *CEP290*-1 (26-X-27; 5)	GCTAAGTACAGG GACATCTTGC	AGACTCCACTTGTTCTTT TAAGGAG	/5HEX/ CTCGTGATCCACC+CGC+CT CG -IABkFQ
c.2991+1655A>G *CEP290*-2 (X-27; 2)	CCCAGTTGTAATT TGTGAAAACATC	TCTTTGCCTTATCCATGC TAGA	/56-FAM/ AG+ACT+CCA+CTTG+TTCTT TTAAGGAG -IABkFQ
c.4723A>T *CEP290* (3)	ATTGCCTGCCACT GCAGAA	TGAAGGTCTTCCTCATG TTTTTA	NA, EvaGreen

+ indicates an LNA base

Commercial TaqMan assays		
Target	*Product name*	*Manufacturer*
GUSB	Hs03929014_g1	ThermoFisher Scientific
HPRT1	Hs02800695_m1	ThermoFisher Scientific
CRX	Hs00230899_m1	ThermoFisher Scientific
NRL	Hs00172997_m1	ThermoFisher Scientific
NR2E3	Hs00183915_m1	ThermoFisher Scientific
AFF4	Hs00969473_m1	ThermoFisher Scientific
ATXN2	Hs01002846_m1	ThermoFisher Scientific
C8orf37	Hs00289484_m1	ThermoFisher Scientific
FANCD2	Hs00395704_m1	ThermoFisher Scientific
INSR	Hs00961560_m1	ThermoFisher Scientific
PTGER3	Hs00988369_m1	ThermoFisher Scientific

Supplementary Table 5. ddPCR program

**One-step ddPCR**		
Step	*Temp*	*Minutes*
1	42 °C	60
2	95°C	10
3	95°C	0.5
4	59°C	1
5	Repeat step 3-4 39x	
6	98°C	10
7	4°C	Indefinite
**Two-step ddPCR**		
Step	*Temp*	*Minutes*
1	95°C	5
2	95°C	0.5
3	60°C	1
4	Repeat step 3-4 39x	
5	4°C	5
6	90°C	10
7	4°C	Indefinite

Supplementary Table 6. Primary antibodies and probes

**Antibody or probe**	**Supplier**	**Dilution**
DAPI (nuclear stain)	Sigma-Aldrich	2 μg/mL
Mouse anti-Pericentrin	Abcam	1:1000
Rabbit anti-Arl13b	ProteinTech	1:1000
Rabbit anti-recoverin	Millipore	1:500
Mouse anti-cone arrestin clone 7G6	kind gift of Wolfgang Baehr	1:100

Supplementary Table 7. In vivo doses and corresponding concentration in the vitreous humor

**Cynomolgus Monkey (Vitreal Volume 1.53 mL)**	
IVT dose	Vitreal Conc.
60 µg	6.22 µM
100 µg	10.37 µM
900 µg	93.37 µM
**Dutch Belted Rabbit (Vitreal Volume 1.12 mL)**	
IVT dose	Vitreal Conc.
100 µg	14.17 µM

Supplementary Table 8. List of differentially expressed gene in LCA10 retinal organoids following 1 μM QR-110 treatment.

Gene ID	Gene Length	Mean Untreated	Mean QR-110	log2Ratio (QR-110 / Untr)	Regulation QR-110 / Untr	Probability	Symbol
100526830	2218	2.59	0.01	-8.017	Down	0.802	SLX1A-SULT1A3
642799	1088	19.585	5.3	-1.886	Down	0.860	NPIPA2
8293	1935	13.675	3.835	-1.834	Down	0.844	SERF1A
4741	3271	46.13	13.47	-1.776	Down	0.870	NEFM
4747	3854	45.55	16.175	-1.494	Down	0.847	NEFL
100874097	443	13.025	4.96	-1.393	Down	0.803	UBE2Q1-AS1
2785	962	33.425	15.885	-1.073	Down	0.802	GNG3
2878	1805	77.88	162.59	1.062	Up	0.814	GPX3
1298	2831	12.375	26.565	1.102	Up	0.801	COL9A2
59	1460.21	23.015	49.85	1.115	Up	0.815	ACTA2
344658	2399.03	13.72	30.41	1.148	Up	0.811	SAMD7
9284	1102	13.385	30.09	1.169	Up	0.813	NPIPA1
1278	5411	65.91	150.74	1.193	Up	0.829	COL1A2
6029	299	23.635	54.24	1.198	Up	0.824	RN7SL1
1281	5490	76.075	175.23	1.204	Up	0.831	COL3A1
3490	1137	8.93	20.81	1.221	Up	0.808	IGFBP7
2719	2329	20.165	50.75	1.332	Up	0.835	GPC3
1277	5927	92.375	233.665	1.339	Up	0.843	COL1A1
2537	837.62	11.09	29.15	1.394	Up	0.833	IFI6
3856	1812.95	5.88	16.695	1.506	Up	0.827	KRT8
10457	2757.64	6.09	17.37	1.512	Up	0.828	GPNMB
2697	3169	13.085	39.775	1.604	Up	0.858	GJA1
6085	102	6.905	23.705	1.779	Up	0.860	RNY3
7980	2444	8.6	31.79	1.886	Up	0.870	TFPI2
9076	3452	5.11	19.105	1.903	Up	0.860	CLDN1
6490	2141.53	7.925	32.335	2.029	Up	0.880	PMEL
245912	189	1.955	8.475	2.116	Up	0.833	DEFB109P1
3294	1451	2.645	11.47	2.117	Up	0.853	HSD17B2
729330	1782	10.15	44.435	2.130	Up	0.887	OC90
4308	4867.98	2.945	13.005	2.143	Up	0.860	TRPM1
646960	2158	1.585	7.715	2.283	Up	0.831	PRSS56
2792	720	1.84	9.25	2.330	Up	0.851	GNGT1
1306	5422	1.605	8.21	2.355	Up	0.843	COL15A1
314	2681	2.27	12.03	2.406	Up	0.869	AOC2
101929127	430.83	1.055	5.925	2.490	Up	0.816	PGM5P3-AS1
7306	2876	2.285	13.405	2.553	Up	0.877	TYRP1
1634	4309.39	2.02	14.665	2.860	Up	0.894	DCN

Gene ID	Gene Length	Mean Untreated	Mean QR-110	log2Ratio (QR-110 / Untr)	Regula-tion QR-110 / Untr	Probability	Symbol
7299	2082	0.755	5.49	2.862	Up	0.826	TYR
22915	4973	0.69	5.185	2.910	Up	0.820	MMRN1
7276	938	1.005	8.825	3.134	Up	0.875	TTR
642778	1098	0.025	6.505	8.023	Up	0.931	NPIPA3
26829	119	0.01	3.115	8.283	Up	0.837	RNU5E-1
6705	671	0.01	3.44	8.426	Up	0.855	SPRR2F
101929829	1666	0.01	3.45	8.430	Up	0.855	LOC101929829

Supplementary Methods

Sequencing

CEP290 c.5668G>T (p.Gly1890X) mutant transcripts were amplified using the AmpliTaq Gold® 360 DNA polymerase kit (Thermo Scientific) with standard cycling conditions and ran on Bioanalyzer (Agilent 2100) to check the size on a DNA1000 chip (Agilent). Primer sequences are summarized in **Supplementary Table 4**. Results were analyzed using Agilent2100 expert software. The PCR product was directly purified using the NucleoSpin® Gel and PCR-clean up (Macherey-Nagel) according to manufacturer’s protocol and sent to BaseClear (Leiden, The Netherlands) for Sanger sequencing.

PBMC viability and stimulation assay

Buffy coats of 5 consenting healthy human blood donors were collected (Sanquin Blood Bank, Leiden, the Netherlands) and PBMC were isolated using Ficoll Paque (GE Healthcare) density gradient centrifugation according to the manufacturer’s instructions within 24 hours after buffycoat extraction. PBMC were cryopreserved in medium containing 10% DMSO and stored at -150˚C until use. Cryopreserved PBMC were thawed and viability was assessed by trypan blue exclusion method. Assay was performed only if the PBMC viability was >95%. PBMC were cultured in 96-well microtiter plates ($1.0*10^5$ per well) for 24 hours in a humidified incubator at 37˚C along with lead candidates 1, 2 and 3 (10nM, 100nM or 1µM each), with positive controls Poly(I:C) (tlr-picw, Invivogen) (1µg/ml), CpG (tlrl-bw006, Invivogen) (10µg/ml), LPS (Tlrl-3pelps, Invivogen) (100ng/ml) and R848 (Invivogen) (1µM), or with saline. All conditions were assayed in triplicate. Cell culture supernatants were collected and stored at -80˚C for cytokine measurement at a later moment. Cytokine levels were measured using the Bio-Plex Pro™ Human Cytokine 17-plex Assay (Bio-Rad) on a Luminex MAGPIX platform (Luminex Corporation) according to the manufacturer’s recommendations. After removal of the supernatant, viability of the PBMC was assessed on the remaining cells of the PBMC stimulation assay using the CellTiter-Blue reagent (Promega) according to the manufacturer’s instructions. Each measurement was normalized against its corresponding saline control. Repeated measures One-way ANOVA with Dunnett’s test for multiple comparisons (compared to saline) was performed on the fold change

values, calculated by normalization against the geometric mean of their corresponding triplicate saline control.

Transcriptome analysis

Samples were sequenced using BGISEQ-500RS (BGI Europe, Denmark). On an average 24 million clean reads per sample were generated and mapped to GRCh38 human reference genome using HISAT/Bowtie2. Raw data and processed files are available at gene expression omnibus (GEO) database repository with the accession number GSE116797.
