# Supplementary material for: Splice-Modulating Oligonucleotide QR-110 Restores CEP290 mRNA and Function in Human c.2991+1655A>G LCA10 Models
Source: Mol Ther Nucleic Acids. 2018 Jul 23;12:730–40. doi: 10.1016/j.omtn.2018.07.010 (PMC6092551; doi:10.1016/j.omtn.2018.07.010)
Supplement: Document S2. Article plus Supplemental Information [file mmc2.pdf]

Molecular Therapy
Nucleic Acids
Original Article

Splice-Modulating Oligonucleotide QR-110 Restores *CEP290* mRNA and Function in Human c.2991+1655A>G LCA10 Models

Kalyan Dulla,[1,4] Monica Aguila,[2,4] Amelia Lane,[2] Katarina Jovanovic,[2] David A. Parfitt,[2] Iris Schulkens,[1] Hee Lam Chan,[1] Iris Schmidt,[1] Wouter Beumer,[1] Lars Vorthoren,[1] Rob W.J. Collin,[3] Alejandro Garanto,[3] Lonneke Duijkers,[3] Anna Brugulat-Panes,[2] Ma'ayan Semo,[2] Anthony A. Vugler,[2] Patricia Biasutto,[1] Peter Adamson,[1,2] and Michael E. Cheetham[2]

[1]ProQR Therapeutics, Leiden, the Netherlands; [2]UCL Institute of Ophthalmology, London, UK; [3]Department of Human Genetics, Donders Institute for Brain, Cognition and Behaviour, Radboud University Medical Center, Nijmegen, the Netherlands

Leber congenital amaurosis type 10 (LCA10) is a severe inherited retinal dystrophy associated with mutations in *CEP290*. The deep intronic c.2991+1655A>G mutation in *CEP290* is the most common mutation in LCA10 individuals and represents an ideal target for oligonucleotide therapeutics. Here, a panel of antisense oligonucleotides was designed to correct the splicing defect associated with the mutation and screened for efficacy and safety. This identified QR-110 as the best-performing molecule. QR-110 restored wild-type *CEP290* mRNA and protein expression levels in *CEP290* c.2991+1655A>G homozygous and compound heterozygous LCA10 primary fibroblasts. Furthermore, in homozygous three-dimensional iPSC-derived retinal organoids, QR-110 showed a dose-dependent restoration of mRNA and protein function, as measured by percentage and length of photoreceptor cilia, without off-target effects. Localization studies in wild-type mice and rabbits showed that QR-110 readily reached all retinal layers, with an estimated half-life of 58 days. It was well tolerated following intravitreal injection in monkeys. In conclusion, the pharmacodynamic, pharmacokinetic, and safety properties make QR-110 a promising candidate for treating LCA10, and clinical development is currently ongoing.

INTRODUCTION

Currently, there is an urgent unmet need for treatment of Leber congenital amaurosis type 10 (LCA10), an autosomal recessive inherited congenital blindness. LCA10 is caused by biallelic mutations in centrosomal protein of 290 kDa (*CEP290*) and is the most common form of LCA.[1] An intronic mutation c.2991+1655A>G (also known as p.Cys998*) is the most frequently occurring *CEP290* mutation, particularly in Europe and the USA, with between 60% and 90% of LCA10 individuals having at least one c.2991+1655A>G allele.[1–5] Unlike other *CEP290* pathogenic mutations, which result in a more severe syndromic presentation, major extra-ocular complications are not reported for patients homozygous or compound heterozygous for the *CEP290* c.2991+1655A>G mutation.[1–3,6]

Studies of patient-derived RNA with the c.2991+1655A>G mutation revealed that a hypomorphic cryptic splice site in intron 26 is introduced by the presence of the mutation.[1,7–9] Consequently, two *CEP290* transcripts are produced: a mutant transcript containing an extra cryptic exon (exon X) of 128 nucleotides that introduces a premature stop codon (p.Cys998*) and a wild-type full-length transcript.[1] The hypomorphic nature of the c.2991+1655A>G allele results in significantly lower levels of wild-type CEP290 protein. CEP290 is essential for the formation and stability of primary cilia.[10–13] The photoreceptor outer segment is a specialized primary cilium that is essential for light detection and photoreceptor survival.[14,15] The outer segment is continually renewed with proteins and lipids synthesized in the inner segment, and is highly reliant on the transport of proteins to the outer segment. Therefore, photoreceptors are particularly vulnerable to disruptions of cilia function.[14,16] This might explain why reduced levels of CEP290 lead to retinal dystrophy.[17–19]

Oligonucleotide-mediated pre-mRNA splice modulation is an established mechanism that has been used to restore *CEP290* mRNA and protein function in LCA10 models. Using antisense oligonucleotides to redirect normal splicing of *CEP290* was first demonstrated in patient-derived fibroblasts and immortalized lymphoblast cells.[7,8] In a later study, induced pluripotent stem cells (iPSCs) derived from fibroblasts from a patient homozygous for the *CEP290* c.2991+1655A>G mutation were used to produce retinal pigment epithelium (RPE) and three-dimensional (3D) retinal organoids,

Received 23 February 2018; accepted 18 July 2018;
https://doi.org/10.1016/j.omtn.2018.07.010.

[4]These authors contributed equally to this work.

Correspondence: Michael E. Cheetham, UCL Institute of Ophthalmology, 11-43 Bath Street, London EC1V 9EL, UK.
E-mail: michael.cheetham@ucl.ac.uk
Correspondence: Peter Adamson, ProQR Therapeutics, Zernikedreef 9, 2333 CK, Leiden, the Netherlands.
E-mail: padamson@proqr.com

This is an open access article under the CC BY license (http://creativecommons.org/licenses/by/4.0/).

which contain a laminated retinal structure with an outer nuclear layer (ONL) of photoreceptor-like cells, to study LCA10 pathogenesis.[9] Interestingly, although all cell types showed reduced levels of full-length *CEP290* mRNA and defects in ciliogenesis, the 3D retinal organoids showed the highest levels of aberrant splicing, with greater defects in cilia formation.[9] This increase in exon X incorporation correlated with the differentiation of photoreceptors and the inclusion of photoreceptor-specific exons in other mRNAs, suggesting a potential basis for the retinal specificity of disease associated with c.2991+1655A>G because of reduced levels of *CEP290* in the retina compared with other tissues.[9] Furthermore, treatment of patient-derived retinal organoids with an exon X-blocking morpholino-oligonucleotide led to reduced levels of mutant transcript and increased the level of wild-type *CEP290* mRNA, with a consequent increase in CEP290 protein expression. There was also a functional improvement in the number and length of photoreceptor cilia and rescue of cilia-associated protein localization, suggesting that this approach could be a viable treatment option for LCA10.[9]

In this study, we describe the development of QR-110, a clinical drug candidate oligonucleotide with potential to restore visual function, or slow vision loss, in patients with LCA10. QR-110 is a single-stranded, fully phosphorothioated, and 2′ *O*-methyl-modified RNA oligonucleotide designed to correct the splicing defect resulting from the *CEP290* c.2991+1655A>G mutation. QR-110 represents a fully optimized oligonucleotide that, as we show here, in both homozygous and compound heterozygous fibroblasts and homozygous retinal organoids carrying the *CEP290* c.2991+1655A>G mutation, has the capacity to significantly restore wild-type *CEP290* mRNA and CEP290 protein, and is associated with increased ciliogenesis. Moreover, QR-110 is effective when used gymnotically on retinal organoids, has good accessibility to the retina following intravitreal (IVT) injection, and demonstrates good tolerability following IVT injection. This makes QR-110 an excellent candidate for clinical development.

RESULTS

Identification of Lead Oligonucleotide Targeting the *CEP290* c.2991+1655A>G Mutation

A total of 29 oligonucleotides were designed using an oligo-walk approach around the exon X sequence and were screened in homozygous *CEP290* c.2991+1665A>G LCA10 patient fibroblasts for reduced aberrant splicing and increased production of wild-type *CEP290* mRNA. Oligonucleotides were also screened by *in silico* methods for lack of secondary structures or immune-stimulatory motifs, and suitability for large-scale manufacturing. This approach identified three best-performing oligonucleotides (leads 1–3) (Figure S1), which were further screened for pro-inflammatory potential using *in vitro* peripheral blood mononuclear cell (PBMC) assay (panel of five independent donors). None of the oligonucleotides had a significant effect on PBMC viability (Figure S2), but PBMC challenge by lead 1 resulted in significant induction of cytokine expression (Figure S3). A mild cytokine response was observed for lead 3 and none for lead 2; therefore, leads 2 and 3 were chosen for *in vivo* tolerability assessment. The rabbit is a highly sensitive species for ocular therapeutics dosed via IVT injection and often develops severe ocular inflammation.[20,21] This exaggerated response is advantageous for the initial immune-inflammatory screening of oligonucleotides. In rabbits, lead 2 was better tolerated than lead 3 (Figure S4A). Finally, tolerability of lead 2 was determined in cynomolgus monkey, the preferred nonclinical toxicological species for ocular therapeutics. Monkeys were given an IVT injection of 60 or 100 μg of oligonucleotide per eye (equivalent to 6 and 10 μM, respectively) and studied for 28 days. This single dose of lead 2 was well tolerated without any significant findings (Figure S4B). In a later monkey study this oligo was studied, in a single dose setting, up to 900 μg of oligonucleotide per eye (equivalent to 93 μM), and was well tolerated at least up to 28 days (Figure S4C). Based on this screening, the lead 2 (QR-110) oligonucleotide emerged as the best-performing molecule and was used for further characterization.

QR-110 Oligonucleotide Treatment Restores *CEP290* mRNA and Protein in Patient Fibroblasts

Fibroblasts from two homozygous *CEP290* c.2991+1665A>G LCA10 patients and from two compound heterozygous LCA10 patients with *CEP290* c.2991+1665A>G in *trans* with c.4723A>T (p.Lys1575*) (compound het 1) and c.5668G>T (p.Gly1890*) (compound het 2) were characterized. The levels of *CEP290* transcripts containing exons 26-27 and X-27 (Figure S5A) were quantified by digital droplet PCR (ddPCR). LCA10 cells expressed significantly reduced levels of wild-type *CEP290* exon 26-27 mRNA compared with control cells (Figures 1A and S5). Transcripts containing exon X were detectable only in the LCA10 cells, and not in the control cells. As expected, the levels of exon X-27 were highest in the homozygous c.2991+1665A>G cells, with lower levels in the compound heterozygous cells (Figure 1B). In the compound heterozygous cells, the levels of mutant c.4723A>T and c.5668G>T *CEP290* transcripts were measured by ddPCR and Sanger sequencing, respectively (Figure S5). The levels of c.4723A>T transcript were substantially lower than the 26-27 transcript in compound het 1, and the levels of c.5668G>T were reduced in cDNA compared with genomic DNA, probably as a result of nonsense-mediated decay due to the presence of premature stop codons in the *trans* alleles (Figure S5).

QR-110 treatment increased *CEP290* wild-type (26-27) levels in both homozygous and compound heterozygous LCA cells in a dose-dependent manner (Figure 1A), with simultaneous reduction in exon X-containing transcripts (X-27) (Figure 1B). The increase in *CEP290* transcript levels was translated into a detectable increase in full-length CEP290 protein levels measured by immunoblotting (Figures 1C and 1D). A scrambled oligonucleotide control of the same length and chemistry had no effect on any of these parameters within the same experiments (Figures 1A–1D). Importantly, QR-110 treatment resulted in an increase in *CEP290* mRNA and protein levels in homozygous LCA10 fibroblasts similar to the control cell line levels, or approximately half that of control cell line levels in compound heterozygous LCA10 fibroblasts (Figures 1A and 1D). The

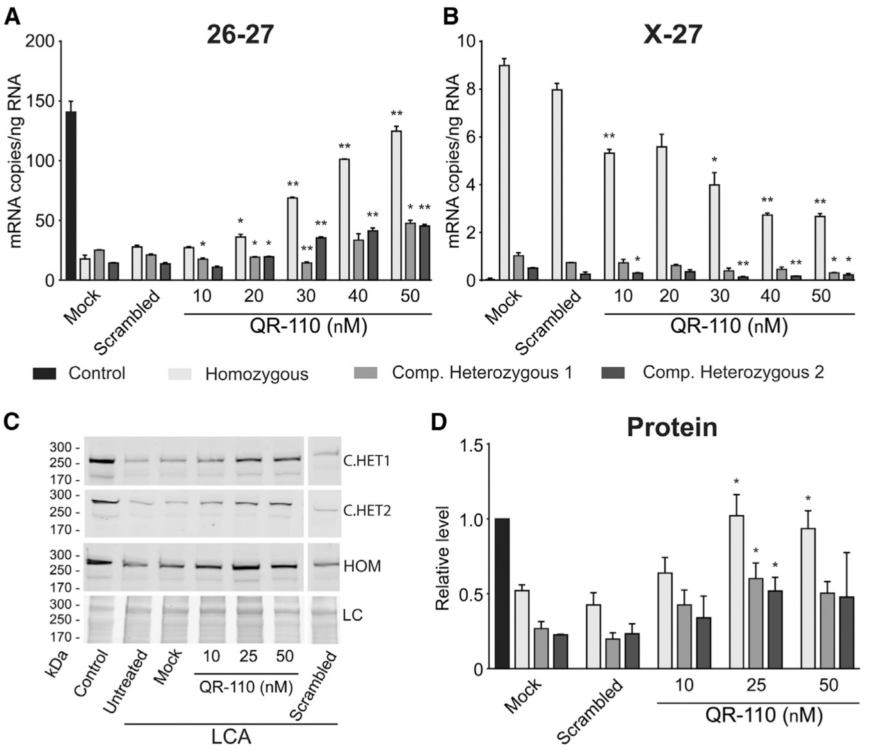

Figure 1. QR-110 Increases Wild-Type *CEP290* mRNA and Protein Levels in c.2991+1655A>G Homozygous and Compound Heterozygous LCA Primary Fibroblasts

(A–D) Quantification of (A) wild-type transcript (exon 26-27), (B) c.2991+1655A>G mutant transcript (exon X-27), and (C and D) protein in control and c.2991+1655A>G homozygous and compound heterozygous primary fibroblasts following treatment with QR-110. Control and LCA cells were treated with different concentrations of QR-110 using PEI-mediated transfection and harvested after 24 (RNA) or 72 hr (proteins). Mock: transfection reagent only; scrambled: different sequence control oligonucleotide with same length and chemistry. mRNA levels were determined by one-step isoform-specific RT-ddPCR. (C) Representative CEP290 immunoblot images from different LCA cell lines. (D) Protein signal was quantified (n = 3 biological replicates per cell line) by densitometry analysis, normalized by protein load (LC), measured by UV illumination of Stain-free gel, and expressed relative to control cell levels. Data are represented as mean ± SEM. *$p < 0.05$; **$p < 0.01$ (Student t test versus mock-treated LCA cells). Compound heterozygous cells had the following genotypes: compound heterozygous 1 (c.2991+1655A>G/c.4723A>T) and compound heterozygous 2 (c.2991+1655A>G/c.5668G>T).

reduced impact of QR-110 on the heterozygous cells was expected because these cells contain only one c.2991+1655A>G allele that can be targeted by QR-110.

QR-110 Photoreceptor Accessibility and Stability *In Vivo*

To test the biodistribution of QR-110 in the eye, biotin-labeled QR-110 and 6-carboxyfluorescein (6-FAM)-labeled QR-110 were used to investigate the ability of QR-110 to reach the photoreceptor ONL, the intended target site for splice correction, after IVT injection in both Dutch-belted rabbits and C57BL/6 mice. The presence of QR-110 was noted in all retinal layers, including the RPE, following IVT injection in both rabbits and mice (Figure 2). The signal was more prominent in the ganglion cell layer and inner nuclear layer, which are closer to the site of injection, and at the outer limiting membrane. This pattern of distribution is consistent with other oligonucleotides administered through the IVT route.[22,23] In rabbit retina, diffuse perinuclear distribution was seen immediately following the IVT injection of biotin-labeled QR-110 and with time developed to an abundant punctate signal (Figure 2A). This indicates a rapid uptake of QR-110 by retinal cells followed by long retention in the retina. This was further supported by a complementary study that quantitatively analyzed QR-110 levels in rabbit retina by hybridization ELISA at different time points following IVT injection (Figure 2B). Linear regression analysis of QR-110 levels in the rabbit retina estimated the retinal half-life at 58 days ($R^2 = 0.87$). 6-FAM-labeled QR-110 showed similar localization in the mouse retina and was also detected at 60 days post-IVT injection (Figure 2C). No retinal staining was observed when PBS or either of the conjugated labels only (without oligonucleotide) was injected at similar molar concentrations (Figures 2A and 2C). Similarly, 6-FAM-labeled QR-110 readily entered the surrogate ONL of control iPSC-retinal organoids and was detected in both the nucleus and the cytoplasm (Figure 2D). These data suggest that QR-110 can access iPSC-derived photoreceptors in 3D organoids following gymnotic treatment, without the need for transfection reagent, which is consistent with their ability to access retinal cells in animals following IVT injection.

QR-110 Treatment Reduces Aberrant Splicing, Increases Wild-Type *CEP290* in Homozygous Retinal Organoids, and Shows No Off-Target Pharmacology

There is no suitable animal model for the c.2991+1665A>G-mediated *CEP290* splicing defect to study the efficacy of QR-110 in retina. Indeed, previous attempts to generate a humanized *CEP290* knockin mouse with the specific c.2991+1655A>G intronic mutation yielded a mouse with no ocular phenotype and limited recognition of the human intronic mutation by the mouse spliceosome.[24,25] Patient iPSC-derived 3D retinal organoids have been used as a relevant test system to show the efficacy of targeting the c.2991+1655A>G intronic mutation.[9] In addition, retinal organoids allow testing of drug candidates in differentiated human photoreceptors, which has a number of unique advantages for studying pathogenic mutations leading to defective splicing, because these mutations may not be recognized by the splicing machinery of other species.[24] Therefore, a 3D retinal organoid model was used for further characterization of the efficacy of QR-110.

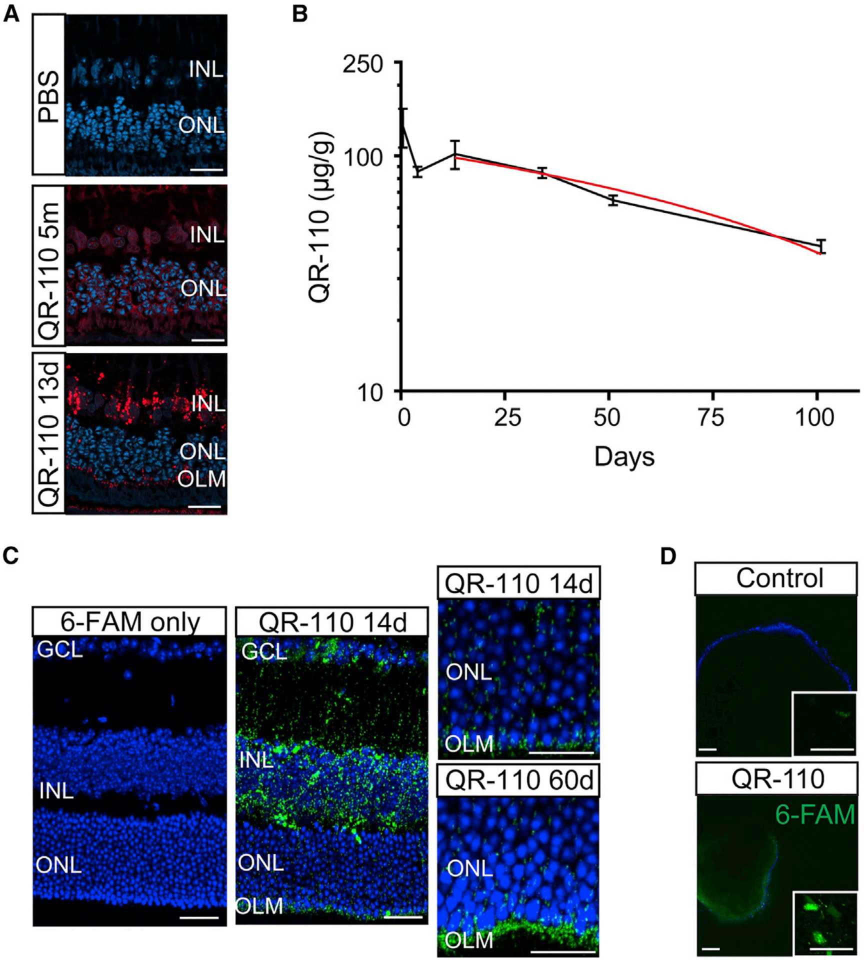

Figure 2. QR-110 Has Good Photoreceptor Accessibility and Stability following Intravitreal Injection

(A) Representative confocal images from Dutch-Belted rabbit retina, at 5 min and 13 days after single IVT of biotin-labeled QR-110 (600 μg). Control animals received PBS and no oligonucleotide. (B) Quantification of QR-110 in Dutch-Belted rabbit retina using hybridization ELISA based on probes complementary to QR-110. Data are represented as mean ± SEM and as microgram of QR-110 per gram of retina. Rabbits were bilaterally dosed (minimum three animals per time point) with a single IVT injection of 100 μg of QR-110 (equivalent to 14.17 μM) and were sacrificed at 0.5, 4, 13, 34, 51, and 101 days. The shape of the QR-110 retinal concentration curve indicates fast uptake and slow elimination, and linear regression analysis (red line) of QR-110 concentration in the elimination phase calculated a retinal half-life of 58 days (R^2 = 0.87). (C) Images of C57BL/6 mouse retina at 14 and 60 days after single IVT injection of 6-FAM-labeled QR-110 (100 μg). Control animals received unconjugated 6-FAM only and no oligonucleotide. (D) Live confocal imaging of retinal organoids, derived from control iPSCs, 48 hr after treatment with 6-FAM-labeled 10 μM QR-110 oligonucleotide (green) or untreated control, counterstained with Hoechst nuclear dye (blue). Larger images show 6-FAM-QR-110 penetrance through all cell layers. Insets show diffuse fluorescence in the cytoplasm and nucleus, as well as some punctate perinuclear staining. Scale bars, 20 μm (A and C); 100 μm (D, main image); 10 μm (D, inset). GCL, ganglion cell layer; INL, inner nuclear layer; OLM, outer limiting membrane; ONL, outer nuclear layer.

Homozygous *CEP290* c.2991+1655A>G iPSCs were produced from patient fibroblasts and characterized, as previously described,[9] and then further differentiated into 3D retinal organoids. Recoverin and cone arrestin immunoreactive cells in the ONL of the retinal organoids and PCR analyses of photoreceptor differentiation markers (*CRX*, *NRL*, and *NR2E3*) confirmed the presence of photoreceptor-like cells and no effect on the expression of these photoreceptor markers following oligonucleotide treatment (Figures 3A–3C). Retinal organoids were gymnotically treated with QR-110. The concentrations of QR-110 were higher than those used in transfected fibroblasts and similar to those used previously for morpholino with Endoporter treatment of retinal organoids.[9]

RT-PCR analysis of QR-110-treated retinal organoids showed significantly increased levels of the correctly spliced, wild-type *CEP290* (exon 26-27) transcript and a reduction in the transcript containing the cryptic exon (26-X-27) (Figures 3D and 3E). Wild-type transcript levels were increased in a dose-dependent manner, reaching more than 90% of the total transcript after treating with 10 μM QR-110. All concentrations of QR-110 led to an increase in the proportion of the wild-type transcript. This was further confirmed by quantitative ddPCR analysis. Increases relative to untreated retinal organoids ranged from 20% at 0.3 μM QR-110 to approximately 140% at 1 or 3 μM QR-110, further increasing to 500% at 10 μM QR-110 (Figure 3F). QR-110 treatment also evoked notable and statistically significant reductions in the cryptic exon transcript (26-X-27) *CEP290*; however, these reductions were not as dose related as the changes in wild-type transcript, with the maximum effect already observed at 3 μM QR-110 (Figure 3G). Similar results were observed in retinal organoids gymnotically treated with lead 3 oligonucleotide, demonstrating that the uptake and distribution are independent of sequence and are likely driven by the phosphorothioate chemistry of the oligonucleotide (Figure S6).

The effect of QR-110 on potential mRNA targets other than *CEP290* in retinal organoids was also investigated. Bioinformatic analyses identified no targets at the mRNA level with full complementarity to the QR-110 sequence; however, some potential off-target locations deep in intronic regions far from exon-intron junctions were identified in pre-mRNAs. *FANCD2* and *C8orf37* were prioritized because they are the top BLAST hits to mRNA

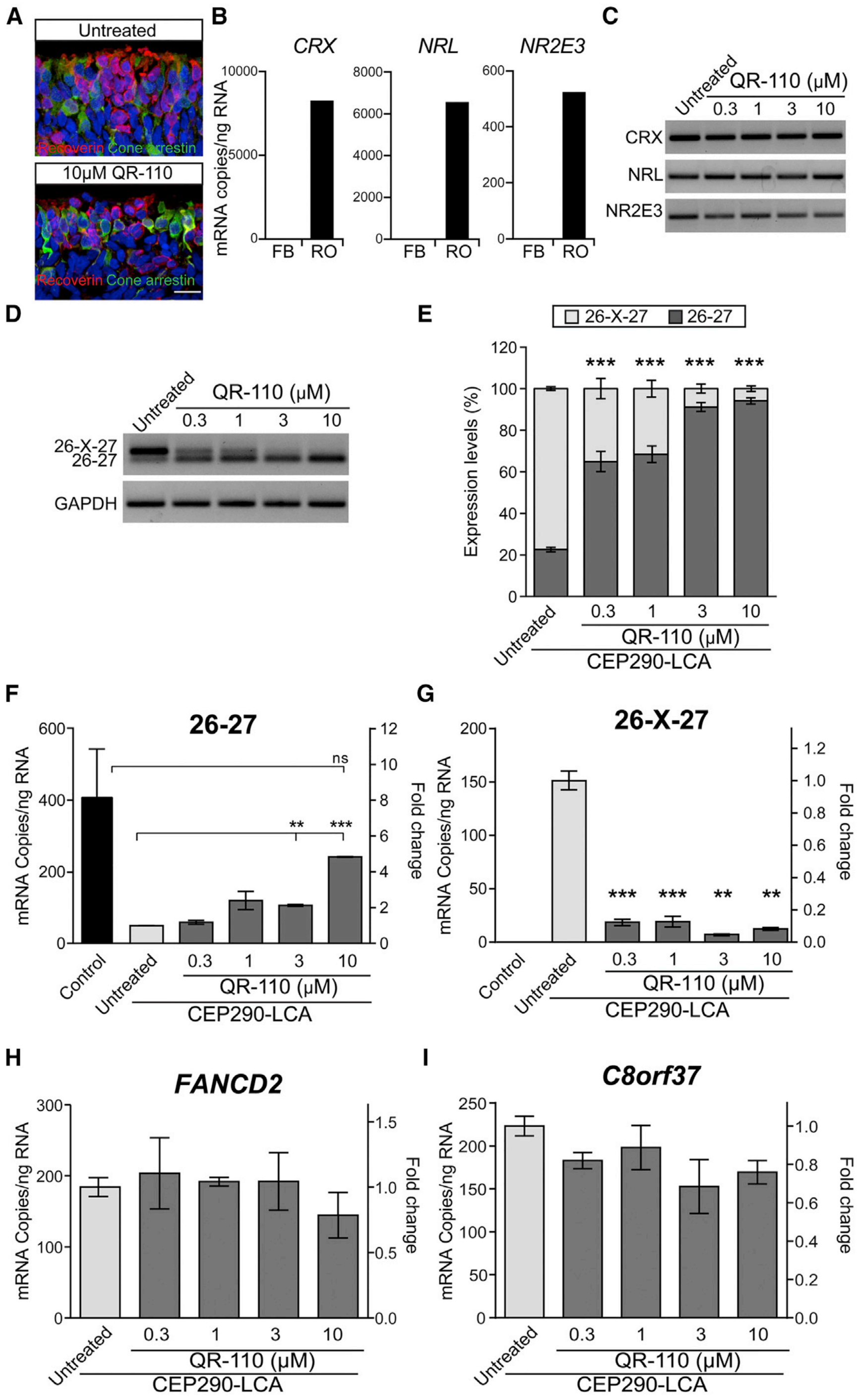

Figure 3. QR-110 Treatment Specifically Increases Wild-Type *CEP290* mRNA in Homozygous LCA Retinal Organoids

Retinal organoids treated with culture medium only (untreated) or culture medium containing different concentrations of QR-110 twice per week, starting from day 96 of maturation, for 4 weeks before assessment at day 124. (A) Recoverin (red) and cone-arrestin (green) staining of retinal organoids used in this study. Scale bar, 10 μm. (B and C) ddPCR quantification (B) and RT-PCR analysis (C) of photoreceptor differentiation marker genes in representative untreated and QR-110-treated homozygous *CEP290* c.2991+1655A>G LCA retinal organoids, at the end of treatment, confirming organoid maturation. FB, fibroblasts; RO, retinal organoids. (D) Representative RT-PCR results of *CEP290* exons 26-27 and 26-X-27. (E) Quantification of bands in homozygous c.2991+1655A>G LCA10 retinal organoids (n = 5–6 retinal organoids per condition) showed a significant increase in wild-type *CEP290* in a dose-response manner, which was further confirmed by isoform-specific RT-ddPCR quantification of *CEP290* (F) wild-type (exon 26-27) and (G) mutant transcript (exon 26-X-27) levels (n = 2–3 per condition). A similar dose response to QR-110 treatment was not observed for potential QR-110 off-targets, (H) *FANCD2* and (I) *C8orf37*, identified by bioinformatics analysis. Gene-specific ddPCR quantification of *FANCD2* and *C8orf37* was investigated in homozygous LCA retinal organoids (n = 2–3 per condition). Statistically insignificant difference (versus untreated) was found for these targets, and no trend of upregulation or downregulation was noticed. Data are represented as mean ± SEM. Student t test versus untreated, $^{**}p < 0.01$; $^{***}p < 0.001$.

with 16/17 base homology to QR-110; furthermore, *C8orf37* was chosen due to its functional relevance in the retina (Figures 3H and 3I).[26] Four additional potential pre-mRNA off-target hits (*AFF4*, *ATXN2*, *CTNNA1*, and *PTGER3*) with a 100% match to QR-110 in their intronic region were selected based on: (1) QR-110 binding distance to the nearest exon, (2) expression level in retina, and (3) the number of PubMed results for terms "gene name" and "eye." The insulin receptor, *INRS*, which has a 16/17 match to QR-110 in the sense strand of the gene sequence, but does not target the mRNA, was also investigated (Figure S7).

Analysis of these off-target effects showed that QR-110 had little or no effect on these targets, highlighting the requirement for full complementarity and/or the presence of important regulatory elements at the site of oligonucleotide binding for on- or off-target activity (Figures 3H, 3I, and S7). It is noteworthy that in these experiments, which illustrated no effect of QR-110 on these potential off-target sequences, QR-110 was effective in correcting the splicing defect in the *CEP290* c.2991+1655A>G mRNA (Figures 3D–3G).

We also performed RNA-sequencing (RNA-seq) to complement this sequence homology-based study with an unbiased approach

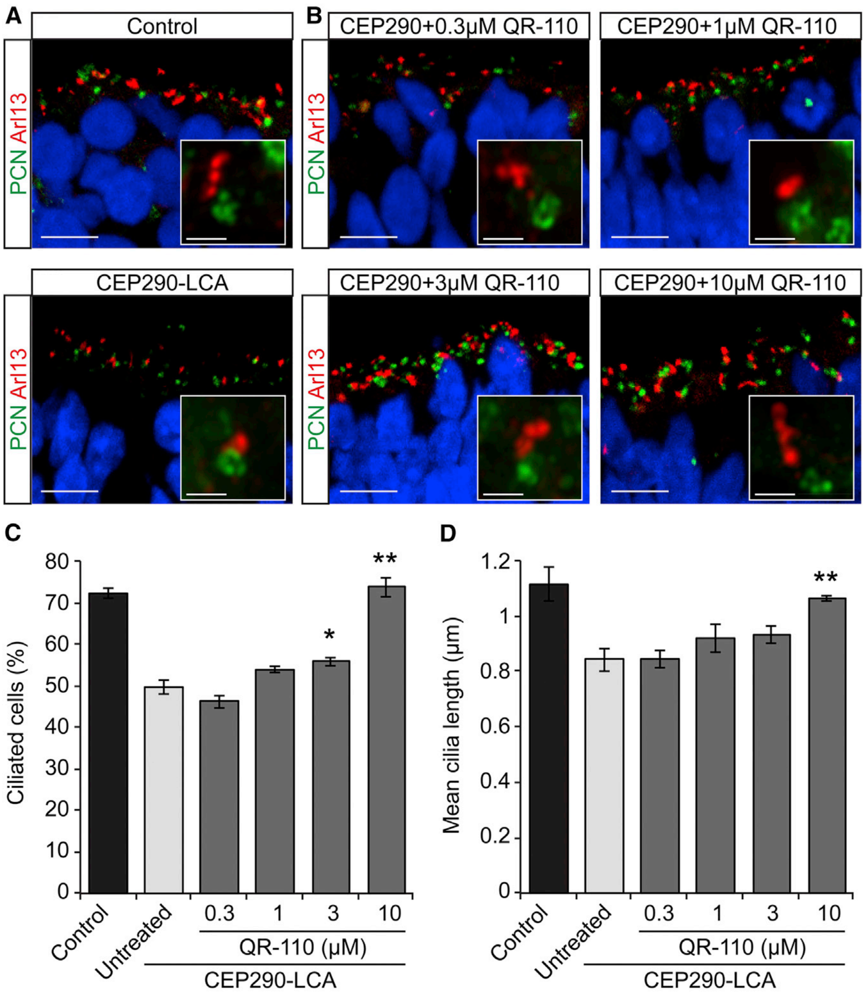

Figure 4. QR-110 Treatment Rescues Ciliation and Increases Cilia Length in c.2991+1655A>G Homozygous LCA Retinal Organoids

Retinal organoids were treated with culture medium only (untreated) or different concentrations of QR-110 from day 96 before assessment at day 124. Representative images of cilia (Arl13, red; pericentrin, green; nuclei, blue) in (A) untreated wild-type control and LCA homozygous c.2991+1655A>G retinal organoids and (B) QR-110-treated LCA homozygous c.2991+1655A>G retinal organoids. Insets show a higher magnification of a cilium. Quantification of cilia (C) incidence and (D) length in retinal organoids treated with 0.3, 1, 3, or 10 μM QR-110. Data are represented as mean ± SEM. Student's t test, versus untreated retinal organoids, *p < 0.05; **p < 0.01. At least 900 basal bodies were scored in each retinal organoid; n = 3 for control and LCA retinal organoids per condition. (A and B) Scale bars, 10 μm (main image); 1 μm (inset).

and identify other potential hybridization-dependent off-targets. Transcriptome analysis was performed to assess the hybridization-dependent off-target effects using two untreated and two 1 μM QR-110-treated LCA10 retinal organoids. This dose was selected because it was effective at suppressing exon X (Figure 3), suggesting effective hybridization and blocking activity, but it did not significantly increase *CEP290* 26-27 mRNA (Figure 3F). Therefore, increased levels of *CEP290* mRNA, such as those observed at higher concentrations of QR-110, which could have wider, downstream "on-target" effects, would not confound the identification of genuine "off-target" effects. Using a 2-fold change as a cutoff, 44 differentially expressed genes were identified, but none has complementarity to QR-110 (Table S8). This number of differential genes in QR-110-treated organoids is similar to transcriptome changes observed for other antisense oligonucleotides and small-molecule drugs.[27] Importantly, none of the seven downregulated genes have been associated with retinal dysfunction. In combination with the selected gene analyses, these data suggest QR-110 does not have any major off-target activity.

QR-110 Treatment Restores Ciliogenesis in *CEP290* Homozygous Retinal Organoids

The reduced levels of CEP290 in LCA10 retinal organoids are associated with defects in photoreceptor ciliation, illustrated by underdeveloped mother centrioles, incompletely formed ciliary vesicles, and membrane-less cilia.[9,19] The effect of QR-110 on ciliogenesis was investigated in *CEP290* c.2991+1655A>G homozygous retinal organoids. Specifically, the number of ciliated cells and the average length of the cilia were assessed (Figure 4). Cilia incidence was measured in the surrogate ONL of retinal organoids by scoring the number of pericentrin-positive basal bodies that were positive for Arl13b (a cilia axoneme marker), and cilia length was measured by quantifying the length of each Arl13b immunopositive axoneme. At low concentrations of QR-110, such as 0.3 and 1 μM, no significant change in either the percentage of ciliated cells or cilia length was observed. In contrast, treatment with 3 μM QR-110 led to a significant increase (~12%) in the percentage of ciliated cells, but the average cilia length remained unchanged; however, 10 μM QR-110 treatment significantly increased both the incidence (~50% increase; Figure 4C) and average length of cilia (27% increase; Figure 4D). At this dose, 74% of the cells were ciliated with an average length over 1 μm, which is a similar cilia incidence and length to the photoreceptor ONL in retinal organoids prepared from healthy control iPSCs (Figures 4C and 4D).

DISCUSSION

The recent success of gene therapy approaches for inherited retinal dystrophies highlights the potential to achieve improved outcomes in these patient populations by targeting the specific underlying genetic defect.[28,29] Large proteins such as CEP290, however, are not deliverable through current AAV gene therapy vectors[30] and require

smaller fragments to be used.[31] Although intronic mutations, such as in *CEP290* c.2991+1655A>G, are an attractive target for gene-editing strategies,[32,33] potential off-target editing, which is irreversible, is a major concern.[34] Furthermore, gene replacement or gene editing would require high levels of transduction to rescue as many cells as possible. Hence, for such indications, oligonucleotide therapeutics provides an attractive alternative. Previous studies have provided *in vitro* proof of concept for the potential of using oligonucleotide-based splice correction therapy for *CEP290* c.2991+1655A>G,[7–9] and the ability of oligonucleotides to reach photoreceptor nuclei *in vivo* following IVT injection.[22,23,35] In addition to potency and delivery, however, a clinical molecule should have a pharmacokinetic profile that supports a feasible dosing regimen, taking into account the ocular administration route, and a safety profile indicative of a positive benefit to risk balance. Therefore, in this study, we have assessed oligonucleotides not only for efficacy, but also for off-target effects, tolerability, and clinical feasibility.

QR-110 oligonucleotide treatment demonstrated a profound concentration-dependent efficacy in both homozygous and compound heterozygous LCA10 fibroblast and LCA10 homozygous retinal organoid models, by mediating splice correction of the exon X-containing *CEP290* mRNA and yielding increased levels of wild-type *CEP290* mRNA. Unlike in fibroblasts, where transfection reagents are required, QR-110 readily entered the surrogate ONL in 3D retinal organoids. This cellular entry of oligonucleotides is proposed to be a feature of oligonucleotides bearing phosphorothioate linkages. The entry of QR-110 into the ONL of the retina was demonstrated in mice using fluorescence labeling and further demonstrated in the rabbit retina with biotin labeling and ELISA. QR-110 is able to penetrate all cellular layers within the retina including the ganglion cell layer, the inner nuclear layer, the ONL, and also the RPE, with the initial exposure in close proximity to the vitreous. Greater exposure of QR-110 to the ONL could potentially be driven by sub-retinal injection or supra-choroidal delivery; however, sub-retinal delivery is a more complicated procedure than IVT injection and unlikely to be used as a regular route of delivery for a reversible therapeutic approach, such as that proposed here. As in the case for other IVT injections and products, so long as there are no adverse consequences of high local concentrations and the target tissue is exposed to a therapeutic level, IVT injections remain a safe and effective route of administration.

QR-110 was shown to be present in the ONL of mouse retina 60 days post-IVT injection, and studies in rabbit suggest an estimated retinal half-life of approximately 58 days, which is consistent with other observations of oligonucleotides with similar chemistry, administered to large eyes by IVT injection.[36] Rabbits are known to be very sensitive to IVT injections,[21] and the IVT injection of 100 μg of QR-110 was associated with a moderate inflammatory reaction. This inflammatory response could have adversely affected the observed half-life, whereas QR-110 was better tolerated in non-human primates. Initial safety assessment of QR-110 was performed in cynomolgus monkey. In a single dose setting of 900 μg per eye, QR-110 was well tolerated for up to 28 days, and no adverse findings related to QR-110 were observed (Figure S4). Therefore, the long half-life of QR-110 should permit infrequent dosing in the range of 3–6 months, which is essential in the clinic given the invasiveness of the IVT injection procedure, but the optimal dosing regimen will need to be determined in clinical studies.

The *CEP290* c.2991+1655A>G homozygous patients comprise only 10%–20% of the LCA10 population, whereas the mutation is reported in compound heterozygosity in >75% of LCA10 patients.[2,37] The clinical presentation of LCA10 is variable and shows an apparent dissociation of structure and function in many patients.[4,38] Optical coherence tomography reveals that almost all patients have central photoreceptor preservation, but vision can range from no light perception to 20/50.[4,38] The reasons for this are currently unclear, and larger detailed studies are needed to better understand any correlation between genotype and phenotype. Nevertheless, the presence of central photoreceptors in most LCA10 patients suggests that their function and survival could be improved by enhanced CEP290 function. Furthermore, LCA10 is a recessive disease with asymptomatic carriers, such that increasing CEP290 levels up to 50% of control CEP290 protein should be sufficient to mediate some functional rescue. Importantly, QR-110 treatment of compound heterozygous fibroblasts increased full-length CEP290 protein to 40%–50% of control levels. Homozygous *CEP290* c.2991+1655A>G retinal organoids have higher levels of aberrant splicing than fibroblasts;[9] nonetheless, the ability of QR-110 to increase the level of wild-type *CEP290* transcript to over 60% of control levels in homozygous retinal organoids with ciliation and cilia length near wild-type control levels suggests that QR-110 could be able to restore sufficient wild-type transcript, even in compound heterozygous photoreceptors.

The use and availability of human patient-derived stem cells that can be differentiated into many different cell types has revolutionized pharmacological approaches to study interventional approaches to inherited disease.[13] In retinal dystrophy, 3D retinal organoids allow the direct study of a human therapeutic, without reference to species-specific molecules, which is particularly important for oligonucleotide therapeutics. Furthermore, the study can be focused on the causative mutation in the relevant genomic context. It has previously been observed that a human knockin transgenic mouse containing the *CEP290* c.2991+1655A>G allele is not as useful as hoped for studying aberrant splicing, because the human gene is processed differently by the mouse photoreceptors and has different splicing enhancer recognition,[24,25] a situation that might occur when trying to model other deep-intronic mutations in this way. Furthermore, human retinal organoids allow retina-specific theoretical off-target effects to be studied, and finally they allow an estimation of the necessary effective vitreal concentration of therapeutic, which can be used to estimate clinical doses in human clinical trials.

Although oligonucleotides are an emerging class of therapeutic agents, retinal diseases such as CMV retinitis and age-related

macular degeneration have already been demonstrated to be amenable to IVT administration of oligonucleotide therapies.[39–41] Vitravene and Macugen, albeit working by different mechanisms of action, are proven oligonucleotide drug therapies for these ophthalmic indications.[42,43] Two antisense oligonucleotides that target pre-mRNA splicing modulation have recently received marketing authorization: Spinraza for spinal muscular atrophy and Exondys51 for Duchenne's muscular dystrophy.[44,45] Oligonucleotide therapeutics targeted at editing pre-mRNA are very selective, with few, if any, off-target effects. They have predicable delivery to all cellular layers of the retina and have a long retinal half-life, allowing for infrequent IVT dosing. Therefore, such therapeutics could be applicable to a wide range of monogenic inherited retinal dystrophies. Importantly, the process of modulating pre-mRNA splicing ensures that the impact of the RNA therapeutic is fully reversible, and that the maximum treatment effect can only be to restore normal levels of target mRNA. This is potentially important in LCA10, because it has been noted previously that overexpression of CEP290 is associated with cellular toxicity.[46] Therapeutic oligonucleotides, such as QR-110, do not pose such a risk, and their excellent safety profiles and efficacy encourage translation to the clinic.

MATERIALS AND METHODS

Animal Care

In-life phase of the rabbit (and monkey) studies were carried out by external contract research organizations (CROs) (Covance Laboratories, USA [rabbit] and Charles River Laboratories, Canada [monkey]) who obtained relevant approvals, and the studies conform to all relevant regulatory standards. The mice used in this study were all female C57BL/6J mice 6–7 weeks of age at the time of purchase from a commercial supplier (Charles River). All procedures on mice were conducted according to the Home Office (UK) regulations, under the Animals (Scientific Procedures) Act of 1986, and with local (UCL-Institute of Ophthamology, London, UK) ethics committee approval. Animals were maintained at constant 12-hr light-dark cycle with access to food and water *ad libitum* for at least 1 week prior to initiating IVT injections, which were performed as described previously.[47] Rabbits used in this study were all males, aged 3–4 months, housed in individual, suspended cages with appropriate enrichment devices. Animals were acclimatized for at least 7 days prior to IVT injection.

Cell Culture and QR-110 Transfection

Fibroblasts (see Table S1) were generated from skin biopsies as previously described.[48] Cell lines were cultured in DMEM-high-glucose AQmedia (Sigma-Aldrich) supplemented with 20% fetal bovine serum (FBS; Biowest, France) and 1% sodium pyruvate (Sigma-Aldrich). Fibroblasts were transfected with oligonucleotides (see Table S2) using polyethyleneimine (PEI) Max transfection reagent (Polysciences, Germany) with an oligo-to-PEI ratio of 1:4 and incubated for 24 hr (RNA analysis) or 3 days (protein analysis) at 37°C. Alternatively, cells were mock treated with transfection reagent only, without oligonucleotide.

3D Retinal Organoids and QR-110 Treatment

iPSCs were generated from c.2991+1655A>G homozygous fibroblasts using three integration-free episomal plasmids from Addgene: pCXLE-hOCT3/4-shp53-F, pCXLE-hUL, and pCXLE-hSK. Reprogramming was performed as described previously.[9,49,50] In brief, 1×10^6 cells were electroporated with 1 μg of each plasmid using the Amaxa Nucleofector I device and cultured on 0.1% gelatin-coated dishes in DMEM (Life Technologies) supplemented with 10% FBS (Lonza), 1 mM non-essential amino acids (NEAA) (Life Technologies), 1 mM GlutaMAX (Life Technologies), 0.5 mM sodium butyrate (Sigma-Aldrich), and 1% penicillin and streptomycin (Life Technologies). After 7 days, the cells were replated at 2×10^5 cells per well into six Geltrex (Life Technologies)-coated wells. The next day, the media were changed to Essential-8 (E8) media (Life Technologies) supplemented with 0.5 mM sodium butyrate for a further 6 days, after which the cells were cultured in E8 media until iPSC colonies appeared (approximately 25 days). Clonal iPSC lines were mechanically isolated and maintained in E8 media.

Directed differentiation of iPSCs into 3D retinal organoids was based on the protocol by Nakano et al.[50] Table S3 summarizes the different media used for differentiation. iPSCs were dissociated using TrypLE (Life Technologies) and plated at a density of 9,000 cells per well in V-shaped 96-well plates in E8 supplemented with 10 μM Y-27632 (Millipore) (day 0). From days 2–14, medium was exchanged every 2 days with embryoid body 1 (EB1) medium and from days 14–20 with EB2 medium every 3–4 days. At day 20, the embryoid bodies were transferred to non-adherent 25-well plates, and EB2 medium was exchanged for neural retinal differentiation (NR) medium every 3–4 days for up to 17 weeks.

LCA homozygous retinal organoids were treated with 0.3, 1, 3, or 10 μM QR-110, diluted in NR medium supplemented with 0.5 μM retinoic acid. A total of eight doses, twice per week, was given from day 96 until day 124. The localization of the QR-110 was assayed following the treatment of wild-type retinal organoids, at day 96, with 10 μM 6-FAM-labeled QR-110 for 48 hr.

RT-PCR

RNA was extracted from the organoids using RNeasy Mini Kit (QIAGEN), and cDNA synthesis was performed using Tetro cDNA synthesis kit (Bioline). *CEP290*, retinal differentiation markers (*CRX*, *NRL*, and *NR2E3*), and *GAPDH* were amplified by PCR using GoTaq Green (Promega) with standard cycling conditions. Primers used for RT-PCR are listed in Table S4. Densitometry analysis of the bands was performed using ImageJ. Student's t test (two-tailed, equal variance) was used for statistical analysis.

Digital Droplet PCR

RNA was isolated from the fibroblasts using the RNeasy Plus Mini kit (QIAGEN). ddPCR was performed, except for the c.4723A>T assay, using the One-Step RT-ddPCR Advanced Kit for Probes (Bio-Rad)

according to the supplier's protocol on the QX200 system (Bio-Rad). Primers and probes used are listed in Table S4. Assays contained 900 nM forward and reverse primer each and 250-nM-labeled probe. The *CEP290* c.4723A>T (p.Lys1575X) assay was performed using two-step ddPCR. First, cDNA was synthesized from 1 μg of RNA using Maxima Reverse Transcriptase and Random Hexamers (Thermo Fisher Scientific) according to the manufacturer's instructions. Then ddPCRs were prepared containing 1× QX200 ddPCR EvaGreen Supermix (Bio-Rad), 900 nM forward and reverse primer each, and 50 ng of cDNA. The ddPCR programs are summarized in Table S5. Each sample was analyzed in duplicate. Absolute quantification was performed in QuantaSoft software (Bio-Rad). The copy number was divided by the amount of input RNA (in nanogram) and normalized to geometric mean of *GUSB* and *HPRT1* (and multiplied by average *GUSB* and *HPRT1* geometric mean of all samples, as a constant). Student's t test (two-tailed, equal variance) was used for statistical analysis.

Western Blotting

Cell pellets were lysed in radioimmunoprecipitation assay (RIPA) protein lysis buffer supplemented with protease inhibitor cocktail (Roche) and further homogenized using a 29G needle. Protein concentrations were determined using the Pierce BCA Protein Assay Kit (Thermo Scientific). The protein samples (80 μg) were loaded onto a Stain-free 4%–15% precast Mini-Protein TGX gel (Bio-Rad), followed by transfer to polyvinylidene fluoride (PVDF) membranes (Millipore). After 1-hr incubation with Odyssey Blocking Buffer (Li-Cor), the membranes were probed overnight with polyclonal rabbit anti-human CEP290 antibody (1:1,000; Novus Biologicals), followed by 1-hr incubation with goat anti-rabbit IRDye 680RD (1:10,000; Li-Cor). The membranes were scanned with the Odyssey CLx Imager (Li-Cor) using Image Studio Lite software. The total protein load (visualized by 1-min UV activation of the protein gel using the GelDoc XR+ system) and the CEP290 protein signal were quantified with Image Studio Lite software. The CEP290 signal was normalized to the total protein load. Student's t test on log2-transformed data was used for statistical analysis.

Cilia Assay and Immunofluorescence

Organoids were removed from culture media and fixed in 4% paraformaldehyde at 4°C for 30 min. Post-fixation retinal organoids were cryoprotected by incubation overnight in 30% sucrose in PBS and then frozen and cyrosectioned. Cryosections were incubated in blocking buffer (3% BSA and 10% normal donkey serum in PBS) for 1 hr at room temperature before incubation with primary antibodies (Table S6) for 2 hr at room temperature. Species-specific anti-IgG Alexa Fluor 488 or 594 secondary antibodies were used, and nuclei were visualized using DAPI (2 μg/mL). Images were obtained using a Carl Zeiss LSM700 laser-scanning confocal microscope, except for the high-magnification images that were obtained using the Airyscan mode on a Carl Zeiss LSM710 microscope. Images were exported from Zen 2009 software and prepared using Adobe Photoshop and Illustrator CS4. All measurements were performed in ImageJ and Adobe Photoshop. For cilia measurements, maximum intensity projections of z stacks were used in the analysis. The incidence of organoid ciliation (%) was determined by counting the total number of pericentrin immunopositive structures associated with Arl13b immunopositive cilia. Cilia length was assessed by measuring Arl13-positive cilia length using Adobe Photoshop. Student's t test (two-tailed, equal variance) was used for statistical analysis.

QR-110 Localization in Retina

Dutch-Belted rabbits were sacrificed 5 min or 13 days after IVT injection of 600 μg of biotin-labeled QR-110. Rabbit eyes were removed and fixed in modified Davidson's fixative, transferred to 70% ethanol, and embedded in paraffin. Tissue sections were deparaffinized, and biotin-labeled QR-110 was visualized using streptavidin-Alexa 647. Images were acquired on an LSM800 confocal microscope (Zeiss).

C57BL/6 mice were sacrificed 14 or 60 days after IVT injection of 100 μg of 6-FAM-labeled QR-110. Mice eyes were fixed overnight at 4°C in 4% paraformaldehyde and transferred to 30% sucrose. Posterior chambers were dissected and lenses removed before embedding and freezing in OCT compound. 6-FAM-labeled QR-110 was directly visualized in cryosections, and images were acquired on an LSM700 confocal microscope (Zeiss).

Wild-type control retinal organoids, at day 96, were treated with 10 μM 6-FAM-QR-110 diluted in NR media or NR media alone and were kept in the dark for the duration of the treatment. After 48-hr incubation, retinal organoids were washed twice with NR media, stained with Hoechst for 20 min, washed with NR media, and imaged live on the Zeiss LSM 510 inverted confocal microscope with heated stage.

QR-110 Quantitation by ELISA

Rabbit retinas were homogenized in 96 μL of extraction buffer (100 mM NaCl, 2 mM $CaCl_2$, 0.25 mM Tris [pH 8.0]) with 0.05% Tween per milligram tissue weight, using a Precellys tissue homogenizer (Bertin Technologies) and Zirconia beads (Biospec Products). Homogenates were incubated with 0.92 mg/mL Proteinase K (Sigma-Aldrich) for 1 hr at 37°C and centrifuged for 3 min at 3,000 rpm at 4°C. The concentration of QR-110 in the supernatants was determined using two-step hybridization ELISA. First, QR-110 was captured by a fully phosphorothioated biotin-labeled probe (complementary to the 3′ end of QR-110) onto a streptavidin-coated MSD plate (Meso Scale Diagnostics). Next, the plate was washed and incubated with a digoxigenin-labeled detection oligonucleotide probe (complementary to the 5′ end of QR-110). Following washing, the plate was incubated with sheep anti-Digoxigenin antibody (Roche), washed and incubated with Sulpho-Tag donkey anti-sheep antibody (Meso Scale Diagnostics), and analyzed on a Meso Scale Sector S600 (Meso Scale Diagnostics). Calibration curves, generated using four-parameter logistic (4PL) curve fit regression (weighing factor = 1/Y2), were used to calculate the concentration of QR-110 in the test samples.

SUPPLEMENTAL INFORMATION

Supplemental Information includes Supplemental Materials and Methods, seven figures, and eight tables and can be found with this article online at https://doi.org/10.1016/j.omtn.2018.07.010.

AUTHOR CONTRIBUTIONS

Conceptualization, P.A. and M.E.C.; Original Draft, K.D.; Writing, M.E.C., P.A., M.A., and K.D.; Resources, R.W.J.C.; Supervision, K.D., W.B., L.V., R.W.J.C., A.A.V., P.B, P.A., and M.E.C.; Investigation and Analyses, M.A., A.L., K.J., I. Schulkens, H.L.C., I. Schmidt, A.B.-P, D.A.P., A.G., L.D., and M.S.; Funding Acquisition, P.B.; Review, Editing & Approval of Manuscript, all authors.

CONFLICTS OF INTEREST

An international patent application has been filed by ProQR Therapeutics (WO 2016/135334) describing methods and means regarding oligonucleotide therapy for Leber congenital amaurosis. P.B. and H.C. are listed as inventors on this application. K.D., I. Schulkens, H.L.C., I. Schmidt, W.B., L.V., P.B., and P.A. are employees of ProQR. M.E.C. has acted as a consultant to ProQR.

ACKNOWLEDGMENTS

We are grateful to the patients for donating tissue for this study. We would like to thank Thomas Hoogenboezem for PBMC assay, Frits van der Ham for *in vivo* delivery work, and Jiayi Miao and Hester Buitendijk for assistance with the fibroblast experiments. We are grateful to Prof. Wolfgang Baehr, University of Utah, for providing 7G6 monoclonal antibody, and to Dr. Frauke Coppieters, University of Ghent, for advice on LCA10 allele frequency. This study was funded by ProQR. iPSC work in the Cheetham lab is also supported by the Wellcome Trust, Fight for Sight, RP Fighting Blindness, and Moorfields Eye Charity.

REFERENCES

1. den Hollander, A.I., Koenekoop, R.K., Yzer, S., Lopez, I., Arends, M.L., Voesenek, K.E., Zonneveld, M.N., Strom, T.M., Meitinger, T., Brunner, H.G., et al. (2006). Mutations in the CEP290 (NPHP6) gene are a frequent cause of Leber congenital amaurosis. Am. J. Hum. Genet. *79*, 556–561.
2. Drivas, T.G., Wojno, A.P., Tucker, B.A., Stone, E.M., and Bennett, J. (2015). Basal exon skipping and genetic pleiotropy: a predictive model of disease pathogenesis. Sci. Transl. Med. *7*, 291ra97.
3. Coppieters, F., Lefever, S., Leroy, B.P., and De Baere, E. (2010). CEP290, a gene with many faces: mutation overview and presentation of CEP290base. Hum. Mutat. *31*, 1097–1108.
4. Sheck, L., Davies, W.I.L., Moradi, P., Robson, A.G., Kumaran, N., Liasis, A.C., Webster, A.R., Moore, A.T., and Michaelides, M. (2018). Leber congenital amaurosis associated with mutations in CEP290, clinical phenotype, and natural history in preparation for trials of novel therapies. Ophthalmology *125*, 894–903.
5. Perrault, I., Delphin, N., Hanein, S., Gerber, S., Dufier, J.L., Roche, O., Defoort-Dhellemmes, S., Dollfus, H., Fazzi, E., Munnich, A., et al. (2007). Spectrum of NPHP6/CEP290 mutations in Leber congenital amaurosis and delineation of the associated phenotype. Hum. Mutat. *28*, 416.
6. den Hollander, A.I., Roepman, R., Koenekoop, R.K., and Cremers, F.P. (2008). Leber congenital amaurosis: genes, proteins and disease mechanisms. Prog. Retin. Eye Res. *27*, 391–419.
7. Collin, R.W., den Hollander, A.I., van der Velde-Visser, S.D., Bennicelli, J., Bennett, J., and Cremers, F.P. (2012). Antisense oligonucleotide (AON)-based therapy for Leber congenital amaurosis caused by a frequent mutation in CEP290. Mol. Ther. Nucleic Acids *1*, e14.
8. Gerard, X., Perrault, I., Hanein, S., Silva, E., Bigot, K., Defoort-Delhemmes, S., Rio, M., Munnich, A., Scherman, D., Kaplan, J., et al. (2012). AON-mediated exon skipping restores ciliation in fibroblasts harboring the common Leber congenital amaurosis CEP290 mutation. Mol. Ther. Nucleic Acids *1*, e29.
9. Parfitt, D.A., Lane, A., Ramsden, C.M., Carr, A.J., Munro, P.M., Jovanovic, K., Schwarz, N., Kanuga, N., Muthiah, M.N., Hull, S., et al. (2016). Identification and correction of mechanisms underlying inherited blindness in human iPSC-derived optic cups. Cell Stem Cell *18*, 769–781.
10. Sayer, J.A., Otto, E.A., O'Toole, J.F., Nurnberg, G., Kennedy, M.A., Becker, C., Hennies, H.C., Helou, J., Attanasio, M., Fausett, B.V., et al. (2006). The centrosomal protein nephrocystin-6 is mutated in Joubert syndrome and activates transcription factor ATF4. Nat. Genet. *38*, 674–681.
11. Kim, J., Krishnaswami, S.R., and Gleeson, J.G. (2008). CEP290 interacts with the centriolar satellite component PCM-1 and is required for Rab8 localization to the primary cilium. Hum. Mol. Genet. *17*, 3796–3805.
12. Sang, L., Miller, J.J., Corbit, K.C., Giles, R.H., Brauer, M.J., Otto, E.A., Baye, L.M., Wen, X., Scales, S.J., Kwong, M., et al. (2011). Mapping the NPHP-JBTS-MKS protein network reveals ciliopathy disease genes and pathways. Cell *145*, 513–528.
13. Tsang, W.Y., Bossard, C., Khanna, H., Peränen, J., Swaroop, A., Malhotra, V., and Dynlacht, B.D. (2008). CP110 suppresses primary cilia formation through its interaction with CEP290, a protein deficient in human ciliary disease. Dev. Cell *15*, 187–197.
14. May-Simera, H., Nagel-Wolfrum, K., and Wolfrum, U. (2017). Cilia—the sensory antennae in the eye. Prog. Retin. Eye Res. *60*, 144–180.
15. Parfitt, D.A., Lane, A., Ramsden, C., Jovanovic, K., Coffey, P.J., Hardcastle, A.J., and Cheetham, M.E. (2016). Using induced pluripotent stem cells to understand retinal ciliopathy disease mechanisms and develop therapies. Biochem. Soc. Trans. *44*, 1245–1251.
16. Wheway, G., Parry, D.A., and Johnson, C.A. (2014). The role of primary cilia in the development and disease of the retina. Organogenesis *10*, 69–85.
17. Chang, B., Khanna, H., Hawes, N., Jimeno, D., He, S., Lillo, C., Parapuram, S.K., Cheng, H., Scott, A., Hurd, R.E., et al. (2006). In-frame deletion in a novel centrosomal/ciliary protein CEP290/NPHP6 perturbs its interaction with RPGR and results in early-onset retinal degeneration in the rd16 mouse. Hum. Mol. Genet. *15*, 1847–1857.
18. Rachel, R.A., Li, T., and Swaroop, A. (2012). Photoreceptor sensory cilia and ciliopathies: focus on CEP290, RPGR and their interacting proteins. Cilia *1*, 22.
19. Shimada, H., Lu, Q., Insinna-Kettenhofen, C., Nagashima, K., English, M.A., Semler, E.M., Mahgerefteh, J., Cideciyan, A.V., Li, T., Brooks, B.P., et al. (2017). In vitro modeling using ciliopathy-patient-derived cells reveals distinct cilia dysfunctions caused by CEP290 mutations. Cell Rep. *20*, 384–396.
20. Short, B.G. (2008). Safety evaluation of ocular drug delivery formulations: techniques and practical considerations. Toxicol. Pathol. *36*, 49–62.
21. de Zafra, C.L.Z., Sasseville, V.G., Matsumoto, S., Freichel, C., Milton, M., MacLachlan, T.K., Farman, C., Raymond, I., Gupta, S., Newton, R., et al. (2017). Inflammation and immunogenicity limit the utility of the rabbit as a nonclinical species for ocular biologic therapeutics. Regul. Toxicol. Pharmacol. *86*, 221–230.
22. Murray, S.F., Jazayeri, A., Matthes, M.T., Yasumura, D., Yang, H., Peralta, R., Watt, A., Freier, S., Hung, G., Adamson, P.S., et al. (2015). Allele-specific inhibition of rhodopsin with an antisense oligonucleotide slows photoreceptor cell degeneration. Invest. Ophthalmol. Vis. Sci. *56*, 6362–6375.
23. Gérard, X., Perrault, I., Munnich, A., Kaplan, J., and Rozet, J.M. (2015). Intravitreal injection of splice-switching oligonucleotides to manipulate splicing in retinal cells. Mol. Ther. Nucleic Acids *4*, e250.
24. Garanto, A., van Beersum, S.E., Peters, T.A., Roepman, R., Cremers, F.P., and Collin, R.W. (2013). Unexpected CEP290 mRNA splicing in a humanized knock-in mouse model for Leber congenital amaurosis. PLoS ONE *8*, e79369.
25. Garanto, A., Duijkers, L., and Collin, R.W. (2015). Species-dependent splice recognition of a cryptic exon resulting from a recurrent intronic CEP290 mutation that causes congenital blindness. Int. J. Mol. Sci. *16*, 5285–5298.
26. Sharif, A.S., Yu, D., Loertscher, S., Austin, R., Nguyen, K., Mathur, P.D., Clark, A.M., Zou, J., Lobanova, E.S., Arshavsky, V.Y., and Yang, J. (2018). C8ORF37 is required for

photoreceptor outer segment disc morphogenesis by maintaining outer segment membrane protein homeostasis. J. Neurosci. *38*, 3160–3176.

27. Hagedorn, P.H., Hansen, B.R., Koch, T., and Lindow, M. (2017). Managing the sequence-specificity of antisense oligonucleotides in drug discovery. Nucleic Acids Res. *45*, 2262–2282.
28. Bennett, J., Wellman, J., Marshall, K.A., McCague, S., Ashtari, M., DiStefano-Pappas, J., Elci, O.U., Chung, D.C., Sun, J., Wright, J.F., et al. (2016). Safety and durability of effect of contralateral-eye administration of AAV2 gene therapy in patients with childhood-onset blindness caused by RPE65 mutations: a follow-on phase 1 trial. Lancet *388*, 661–672.
29. Edwards, T.L., Jolly, J.K., Groppe, M., Barnard, A.R., Cottriall, C.L., Tolmachova, T., Black, G.C., Webster, A.R., Lotery, A.J., Holder, G.E., et al. (2016). Visual acuity after retinal gene therapy for choroideremia. N. Engl. J. Med. *374*, 1996–1998.
30. Wu, Z., Yang, H., and Colosi, P. (2010). Effect of genome size on AAV vector packaging. Mol. Ther. *18*, 80–86.
31. Zhang, W., Li, L., Su, Q., Gao, G., and Khanna, H. (2018). Gene therapy using a miniCEP290 fragment delays photoreceptor degeneration in a mouse model of Leber congenital amaurosis. Hum. Gene Ther. *29*, 42–50.
32. Burnight, E.R., Gupta, M., Wiley, L.A., Anfinson, K.R., Tran, A., Triboulet, R., Hoffmann, J.M., Klaahsen, D.L., Andorf, J.L., Jiao, C., et al. (2017). Using CRISPR-Cas9 to generate gene-corrected autologous iPSCs for the treatment of inherited retinal degeneration. Mol. Ther. *25*, 1999–2013.
33. Ruan, G.X., Barry, E., Yu, D., Lukason, M., Cheng, S.H., and Scaria, A. (2017). CRISPR/Cas9-mediated genome editing as a therapeutic approach for Leber congenital amaurosis 10. Mol. Ther. *25*, 331–341.
34. Nakajima, K., Kazuno, A.A., Kelsoe, J., Nakanishi, M., Takumi, T., and Kato, T. (2016). Exome sequencing in the knockin mice generated using the CRISPR/Cas system. Sci. Rep. *6*, 34703.
35. Garanto, A., Chung, D.C., Duijkers, L., Corral-Serrano, J.C., Messchaert, M., Xiao, R., Bennett, J., Vandenberghe, L.H., and Collin, R.W. (2016). In vitro and in vivo rescue of aberrant splicing in CEP290-associated LCA by antisense oligonucleotide delivery. Hum. Mol. Genet. *25*, 2552–2563.
36. Henry, S.P., Miner, R.C., Drew, W.L., Fitchett, J., York-Defalco, C., Rapp, L.M., and Levin, A.A. (2001). Antiviral activity and ocular kinetics of antisense oligonucleotides designed to inhibit CMV replication. Invest. Ophthalmol. Vis. Sci. *42*, 2646–2651.
37. McAnany, J.J., Genead, M.A., Walia, S., Drack, A.V., Stone, E.M., Koenekoop, R.K., Traboulsi, E.I., Smith, A., Weleber, R.G., Jacobson, S.G., and Fishman, G.A. (2013). Visual acuity changes in patients with leber congenital amaurosis and mutations in CEP290. JAMA Ophthalmol. *131*, 178–182.
38. Jacobson, S.G., Cideciyan, A.V., Sumaroka, A., Roman, A.J., Charng, J., Lu, M., Choi, W., Sheplock, R., Swider, M., Kosyk, M.S., et al. (2017). Outcome measures for clinical trials of Leber congenital amaurosis caused by the intronic mutation in the CEP290 gene. Invest. Ophthalmol. Vis. Sci. *58*, 2609–2622.
39. Stein, C.A., and Castanotto, D. (2017). FDA-approved oligonucleotide therapies in 2017. Mol. Ther. *25*, 1069–1075.
40. Lundin, K.E., Gissberg, O., and Smith, C.I. (2015). Oligonucleotide therapies: the past and the present. Hum. Gene Ther. *26*, 475–485.
41. Gerard, X., Garanto, A., Rozet, J.M., and Collin, R.W. (2016). Antisense oligonucleotide therapy for inherited retinal dystrophies. Adv. Exp. Med. Biol. *854*, 517–524.
42. de Smet, M.D., Meenken, C.J., and van den Horn, G.J. (1999). Fomivirsen—a phosphorothioate oligonucleotide for the treatment of CMV retinitis. Ocul. Immunol. Inflamm. *7*, 189–198.
43. Gragoudas, E.S., Adamis, A.P., Cunningham, E.T., Jr., Feinsod, M., and Guyer, D.R.; VEGF Inhibition Study in Ocular Neovascularization Clinical Trial Group (2004). Pegaptanib for neovascular age-related macular degeneration. N. Engl. J. Med. *351*, 2805–2816.
44. Finkel, R.S., Mercuri, E., Darras, B.T., Connolly, A.M., Kuntz, N.L., Kirschner, J., Chiriboga, C.A., Saito, K., Servais, L., Tizzano, E., et al.; ENDEAR Study Group (2017). Nusinersen versus sham control in infantile-onset spinal muscular atrophy. N. Engl. J. Med. *377*, 1723–1732.
45. Mendell, J.R., Rodino-Klapac, L.R., Sahenk, Z., Roush, K., Bird, L., Lowes, L.P., Alfano, L., Gomez, A.M., Lewis, S., Kota, J., et al.; Eteplirsen Study Group (2013). Eteplirsen for the treatment of Duchenne muscular dystrophy. Ann. Neurol. *74*, 637–647.
46. Burnight, E.R., Wiley, L.A., Drack, A.V., Braun, T.A., Anfinson, K.R., Kaalberg, E.E., Halder, J.A., Affatigato, L.M., Mullins, R.F., Stone, E.M., and Tucker, B.A. (2014). CEP290 gene transfer rescues Leber congenital amaurosis cellular phenotype. Gene Ther. *21*, 662–672.
47. Semo, M., Gias, C., Ahmado, A., Sugano, E., Allen, A.E., Lawrence, J.M., Tomita, H., Coffey, P.J., and Vugler, A.A. (2010). Dissecting a role for melanopsin in behavioural light aversion reveals a response independent of conventional photoreception. PLoS ONE *5*, e15009.
48. Schwarz, N., Carr, A.J., Lane, A., Moeller, F., Chen, L.L., Aguilà, M., Nommiste, B., Muthiah, M.N., Kanuga, N., Wolfrum, U., et al. (2015). Translational read-through of the RP2 Arg120stop mutation in patient iPSC-derived retinal pigment epithelium cells. Hum. Mol. Genet. *24*, 972–986.
49. Okita, K., Matsumura, Y., Sato, Y., Okada, A., Morizane, A., Okamoto, S., Hong, H., Nakagawa, M., Tanabe, K., Tezuka, K., et al. (2011). A more efficient method to generate integration-free human iPS cells. Nat. Methods *8*, 409–412.
50. Nakano, T., Ando, S., Takata, N., Kawada, M., Muguruma, K., Sekiguchi, K., Saito, K., Yonemura, S., Eiraku, M., and Sasai, Y. (2012). Self-formation of optic cups and storable stratified neural retina from human ESCs. Cell Stem Cell *10*, 771–785.

OMTN, Volume *12*

Supplemental Information

Splice-Modulating Oligonucleotide QR-110 Restores *CEP290* mRNA and Function in Human c.2991+1655A>G LCA10 Models

Kalyan Dulla, Monica Aguila, Amelia Lane, Katarina Jovanovic, David A. Parfitt, Iris Schulkens, Hee Lam Chan, Iris Schmidt, Wouter Beumer, Lars Vorthoren, Rob W.J. Collin, Alejandro Garanto, Lonneke Duijkers, Anna Brugulat-Panes, Ma'ayan Semo, Anthony A. Vugler, Patricia Biasutto, Peter Adamson, and Michael E. Cheetham

Supplemental Information

Supplementary Figures

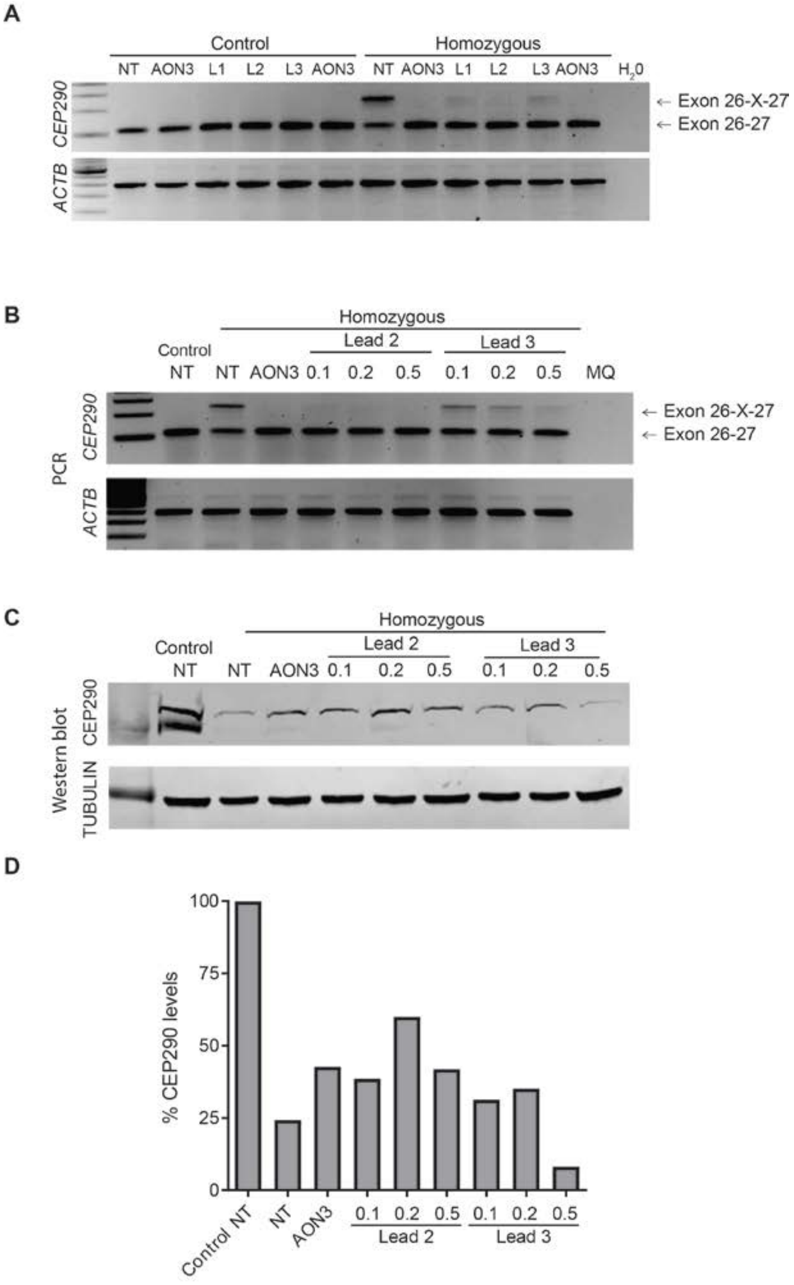

Figure S1. Screening of splice modulating oligonucleotides. (A) Control and c.2991+1655A>G homozygous fibroblasts were transfected with 100 nM each of Lead 1, 2 (i.e. QR-110), 3 or AON3 (Collin et al 2012) oligonucleotide, using Fugene reagent and analyzed for *CEP290* by RT-PCR. Oligo concentrations used here are higher than those of experiments described in the main text due to the use of different transfection reagent. **(B)** Homozygous fibroblasts were treated with 3 concentrations (0.1, 0.2, 0.5 μM) of Lead 2 (i.e. QR-110) or Lead 3 and one concentration (100 nM) of control oligo using Fugene reagent and *CEP290* transcripts were analyzed by RT-PCR and (**C**) CEP290 levels by Western blot. **(D)** Protein signal was quantified by densitometry analysis, normalized by tubulin. NT not treated. n=1. At highest oligo concentration, a reduction in the CEP290 levels was noticed, probably due to cytotoxicity.

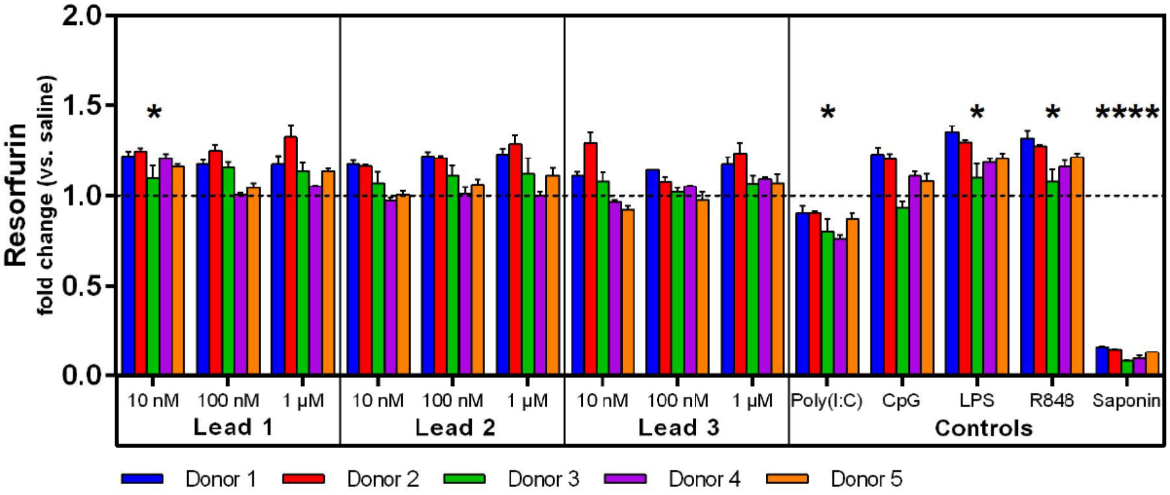

Figure S2. Viability assay of human PBMC treated with lead oligos. Relative number of viable PBMC expressed as fold change of Resorufin fluorescence compared to saline treated PBMC after 24 hours exposure to Lead 1 to 3 (10nM, 100nM or 1µM) or the positive controls Poly(I:C) (1µg/ml), CpG (10µg/ml), LPS (100ng/ml) and R848 (1µM). For all individual biological replicates, fold changes were calculated by normalizing measured RFU against geometric mean of corresponding triplicate saline control. Results are shown per individual donor as the mean+SEM of the triplicate fold change, normalized against the mean of its corresponding saline control (dotted line). Repeated measures One-way ANOVA with Dunnett test for multiple corrections (compared to saline) was performed on the fold change values. Statistical significance is depicted as *(p>0.05) or **** (p>0.0001).

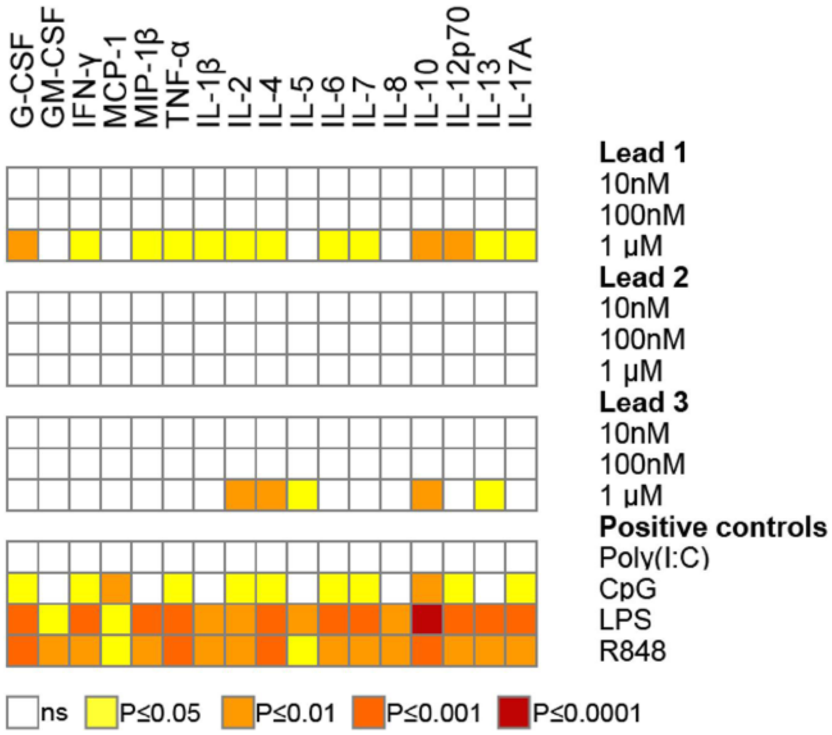

Figure S3. Cytokine production by human PMBC treated with lead oligos. Heat map depicting the statistical significance of cytokine changes compared to saline-treated PBMC, in culture supernatant 24 hours after stimulation of PBMC with Lead 1 to 3 (10nM, 100nM or 1μM) or the positive controls Poly(I:C) (1μg/ml), CpG (10μg/ml), LPS (100ng/ml) and R848 (1μM). Geometric mean of the five human donors with triplicate measurements each. P-values were determined using the Friedman test with Dunn's post-hoc test. Not-significant: ns.

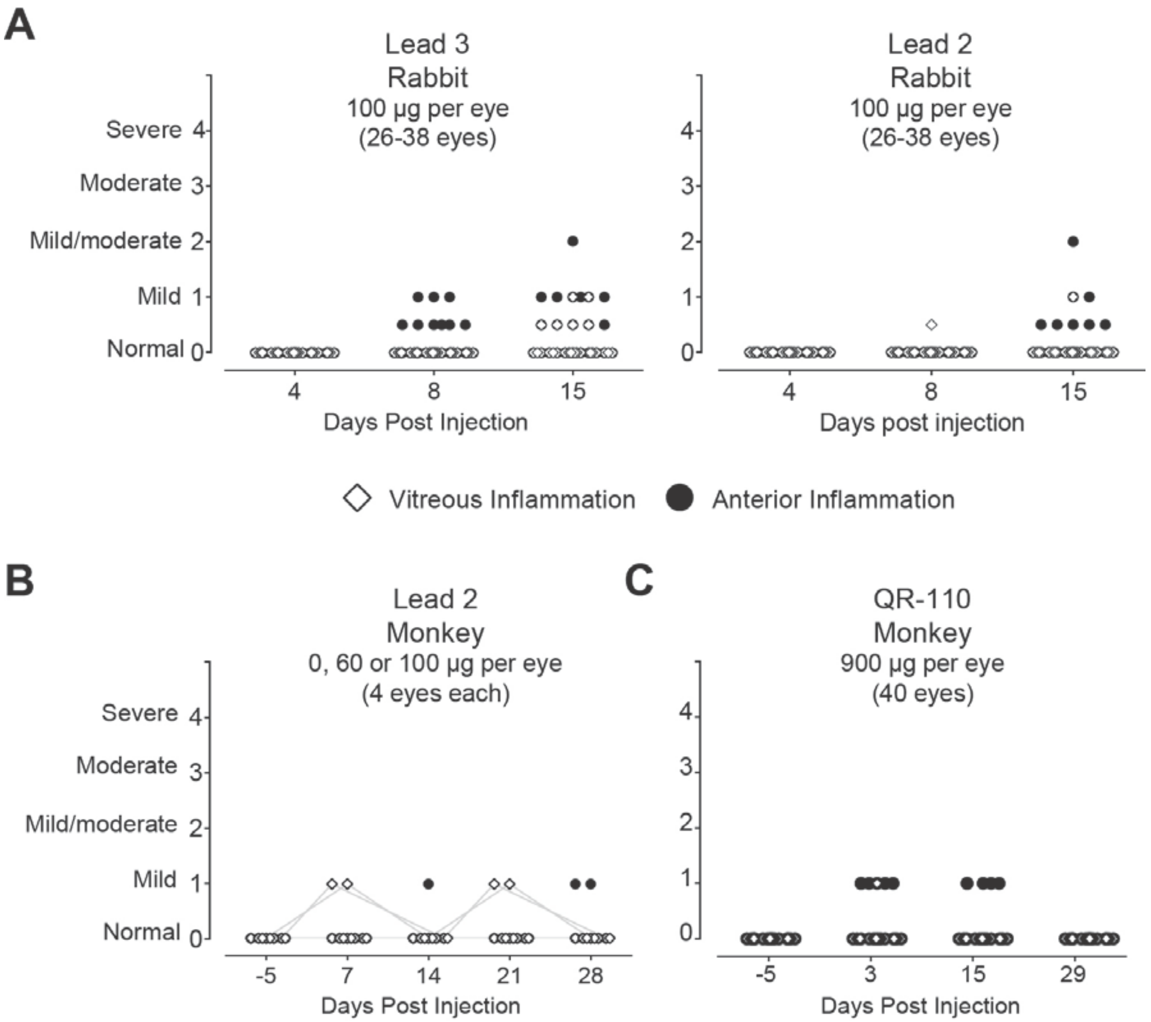

Figure S4. In-vivo tolerability studies. Animals received a single intravitreal injection of test article or just the PBS vehicle. Ophthalmic examinations were performed by a veterinary ophthalmologist using slitlamp biomicroscope and indirect/direct ophthalmoscopy and inflammation, if any, was scored in anterior and posterior segments. (**A**) Dutch-Belted rabbits received a single intravitreal injection of 100 µg (14 µM) per eye of either Lead 2 or Lead 3 oligonucleotide and were assessed on day 4, 8 and 15. Six animals each were sacrificed at each assessment time point for further analysis thus different number of eyes (38-26) were assessed at each time point. (**B**) Cynomolgus monkeys received a single intravitreal injection of either 0, 60 (6 µM) or 100 µg (10 µM) per eye (2 animals per dose level) and the inflammation was assessed. The findings from the animals treated with either 60 or 100 µg are plotted together. Findings from the control animals are connected by the grey lines which highlight the absence of any trend in inflammation in oligo treated animals and only minor fluctuations were seen. (**C**) In a separate study, cynomolgus monkeys were dosed with 900 (93 µM) µg QR-110 per eye and were assessed at 5 days pre-dosing and 3, 15 and 29 days post-posting. *In vivo* doses and the corresponding concentration in the vitreous humor are shown in Table S7.

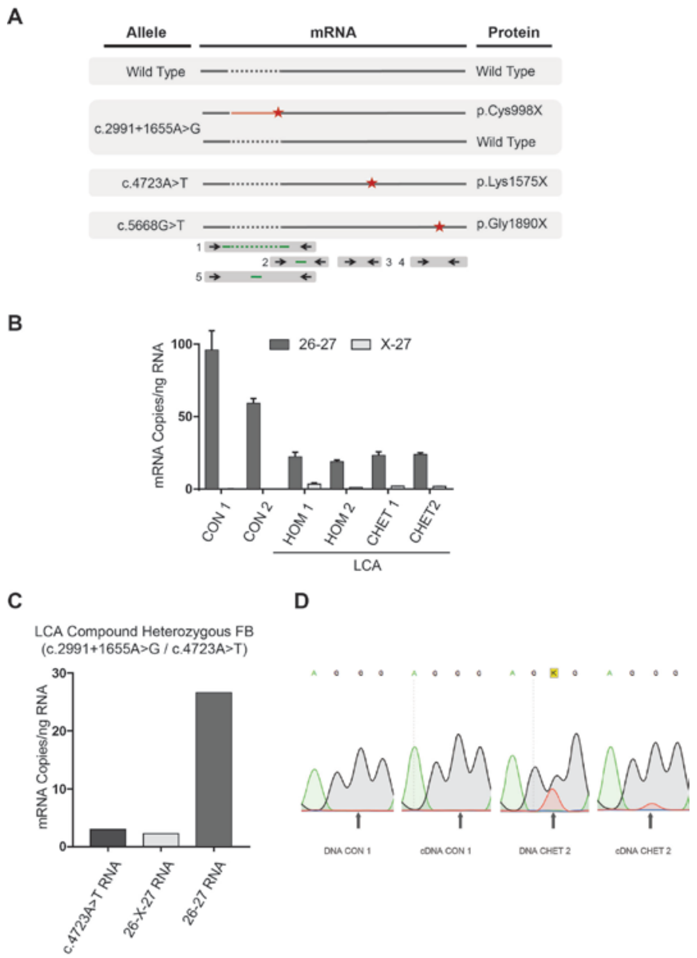

Figure S5. *CEP290* levels are reduced in c.2991+1655A>G homozygous and compound heterozygous LCA patient fibroblasts and the mutant *CEP290* transcripts are degraded by NMD. (**A**) Schematic representation of different *CEP290* alleles present in the LCA models investigated in this study and the corresponding mRNA and protein produced. All alleles produce one type of mRNA apart from the c.2991+1655A>G which produces both wild-type and mutant transcripts. PCR assay design strategy to specifically detect different transcripts is shown at the bottom of the schematic. Wild-type PCR assay (1) relies on the junction of exon 26-27, so this assay also detects transcripts containing c.4723A>T and c.5668G>T. The c.2991+1655A>G PCR assays X-27 (2) and 26-X-27 (5), and c.4723A>T PCR assay (3) are specific for the mutations and do not detect any other transcript. The c.5668G>T assay (4) amplifies the region around the c.5668G>T mutation and sequencing was used to specifically detect the c.5668G>T isoform. The position of stop codons is shown by a red star, PCR primers by arrows and ddPCR probe positions are shown in green**.** (**B**) Wild-type and c.2991+1655A>G *CEP290* expression is measured using ddPCR in two healthy control (CON); and two homozygous (HOM) and two compound heterozygous (CHET) fibroblast cell lines isolated from LCA patients. Control cells expressed wild-type *CEP290* exclusively. In LCA cells c.2991+1655A>G transcripts were only 10% of the wild-type transcripts levels, but wild-type transcripts levels themselves were 23%-36% of the healthy control cells. (**C**) Three different *CEP290* transcripts expressed in the compound heterozygous cell line 1 (c.2991+1655A>G / c.4723A>T) were quantified using isoform specific ddPCR assays. High wild-type levels with significantly low c.4723A>T levels comparable to c.2991+1655A>G transcripts point to NMD mediated degradation of transcripts with the premature stop codon. (**D**) PCR primers binding to exon 41 were used to amplify the region around the c.5668G>T mutation from DNA and cDNA of control (CON) and compound heterozygous (c.2991+1655A>G / c.5668G>T) fibroblasts and sequenced using Sanger sequencing. No c.5668G>T signal was detected in control cells (highlighted with an arrow). In compound heterozygous cells, as expected the c.5668G>T mutation was ~50% of the DNA, whereas the levels were much lower in cDNA, indicating NMD of the c.5668G>T transcript.

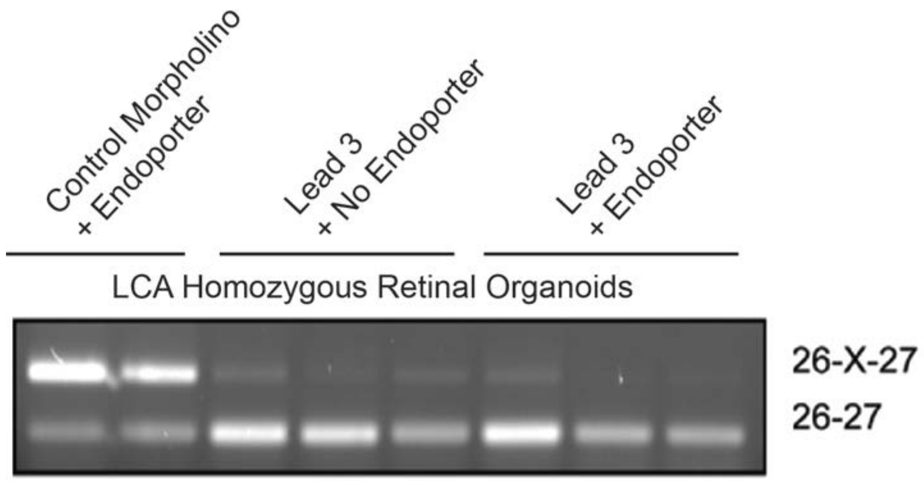

Figure S6. Lead 3, a *CEP290*-directed 2' O methyl phosphorothioate oligo, demonstrates efficacy with and without transfection reagent. Homozygous LCA10 retinal organoids were treated with culture medium containing 10 µM control morpholino (Parfitt et al 2016) with Endoporter, Lead 3 with Endoporter or Lead 3 gymnotically twice per week, starting from day 96 of maturation, for 4 weeks and *CEP290* transcripts were analyzed by RT-PCR.

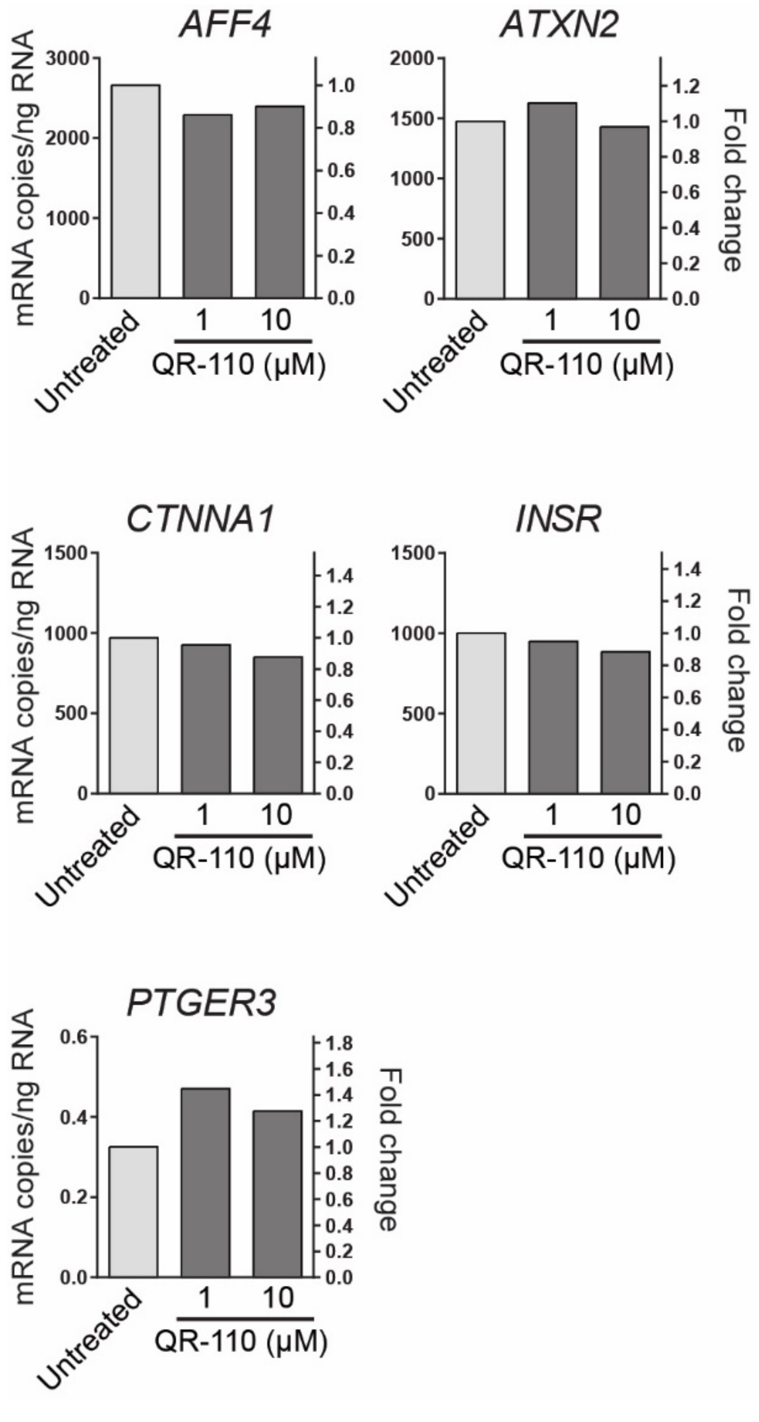

Figure S7. QR-110 effect on a set of potential off-targets investigated in homozygous LCA10 retinal organoids. Transcript levels were determined by gene-specific one-step RT-ddPCR. Results were normalized using housekeeping genes (*GUSB*, *HPRT*) and as expressed as copies per ng of RNA input. All values were expressed as mean ± SEM (one retinal organoid per treatment condition).

Tables

Supplementary Table 1. Cell lines

Name	Genotype	Description
CON1	Wild-type	Control fibroblasts
CON2	Wild-type	Control fibroblasts
HOM1	c.2991+1655A>G / c.2991+1655A>G	p.Cys998X homozygous fibroblasts
HOM2	c.2991+1655A>G / c.2991+1655A>G	p.Cys998X homozygous fibroblasts
CHET1	c.2991+1655A>G / c.4723A>T	p.Cys998X/p.Lys1575X compound heterozygous fibroblasts
CHET2	c.2991+1655A>G / c.5668G>T	p.Cys998X/p.Gly1890X compound heterozygous fibroblasts
HOM3 RO	c.2991+1655A>G / c.2991+1655A>G	p.Cys998X homozygous retinal organoids

Supplementary Table 2. Details of the oligonucleotides used.

Name	Sequence (5’ → 3’)	Manufacturer	Chemistry
QR-110	GGUGGAUCACGAGUUCA	Biospring	Phosphorothioated backbone and 2' O-methyl RNA bases
Lead 1	GGCUCACAUCUGUAAUC	Axolabs GmbH	
Lead 2 (QR-110)	GGUGGAUCACGAGUUCA	Axolabs GmbH	
Lead 3	GGAUAGGUAUGAGAUAC	Axolabs GmbH	
Scrambled	AUAGUAACGGAUUGAGG	IDT DNA	

Supplementary Table 3. Different media used for organoid differentiation.

Name	Contents
Fibroblast culture medium	DMEM – high glucose AQmedia (Sigma-Aldrich), 20% fetal bovine serum (Biowest),1% sodium pyruvate (Sigma-Aldrich)
Embryoid body (EB) medium	Glasgow’s MEM (GMEM, Life Technologies), 20% KOSR (Life Technologies), 1mM NEAA, 1mM sodium pyruvate, 100μM β-mercaptoethanol
EB1 medium	Glasgow’s MEM (GMEM, Life Technologies), 20% KnockOut Serum Replacement (KOSR, Life Technologies), 1mM NEAA, 1mM sodium pyruvate, 100μM β-mercaptoethanol, 10μM Y-27632, 3μM IWR1e (Calbiochem), 2% Matrigel (Corning)
EB2 medium	GMEM, 20% KOSR, 1mM NEAA, 1mM sodium pyruvate, 100μM β-mercaptoethanol, 10% FBS, 1% Matrigel, 10μM Y-27632, 100nM smoothened agonist (SAG; Enzo Life Sciences)
Neural retinal differentiation (NR) medium	DMEM-F12, 10% FBS, 1% N2 supplement (Life Technologies), 0.5μM retinoic acid (RA; Tocris)

Supplementary Table 4. Primers and probes used for RT-PCR and ddPCR.

**RT-PCR assays**		
**Gene name**	**Forward primer**	**Reverse primer**
CEP290	TGCTAAGTACAGGGACATCTTGC	AGACTCCACTTGTTCTTTTAAGGAG
CRX	CCCACTATTCTGTCAACGCCT	TCTTGGCAAACAGTGCCTCC
NRL	CACTGACCACATCCTCTCGG	GAGGGTTCCCGCTTTACCTC
NR2E3	TGGTCCTCTTCAAGCCAGAGA	TTTCACCTCCACCCCCACTA
GAPDH	TGCACCACCAACTGCTTAG	GGATGCAGGGATGATGTTC
c.5668G>T *CEP290* (4)	GGCAAACCCCTGACAGATAA	GGTTTTAGGTCTACTTCCTC

**Custom-designed ddPCR assays**			
Gene name (Fig. S5A)	Forward primer	Reverse primer	Probe
Wild-Type *CEP290* (1)	GCTAAGTACAGG GACATCTTGC	AGACTCCACTTGTTCTTT TAAGGAG	/56-FAM/ CTGGAGT+GT+GA+A+AA+C A+TC -IABkFQ
c.2991+1655A>G *CEP290*-1 (26-X-27; 5)	GCTAAGTACAGG GACATCTTGC	AGACTCCACTTGTTCTTT TAAGGAG	/5HEX/ CTCGTGATCCACC+CGC+CT CG -IABkFQ
c.2991+1655A>G *CEP290*-2 (X-27; 2)	CCCAGTTGTAATT TGTGAAAACATC	TCTTTGCCTTATCCATGC TAGA	/56-FAM/ AG+ACT+CCA+CTTG+TTCTT TTAAGGAG -IABkFQ
c.4723A>T *CEP290* (3)	ATTGCCTGCCACT GCAGAA	TGAAGGTCTTCCTCATG TTTTTA	NA, EvaGreen

+ indicates an LNA base

**Commercial TaqMan assays**		
Target	*Product name*	*Manufacturer*
GUSB	Hs03929014_g1	ThermoFisher Scientific
HPRT1	Hs02800695_m1	ThermoFisher Scientific
CRX	Hs00230899_m1	ThermoFisher Scientific
NRL	Hs00172997_m1	ThermoFisher Scientific
NR2E3	Hs00183915_m1	ThermoFisher Scientific
AFF4	Hs00969473_m1	ThermoFisher Scientific
ATXN2	Hs01002846_m1	ThermoFisher Scientific
C8orf37	Hs00289484_m1	ThermoFisher Scientific
FANCD2	Hs00395704_m1	ThermoFisher Scientific
INSR	Hs00961560_m1	ThermoFisher Scientific
PTGER3	Hs00988369_m1	ThermoFisher Scientific

Supplementary Table 5. ddPCR program

**One-step ddPCR**		
Step	*Temp*	*Minutes*
1	42 °C	60
2	95°C	10
3	95°C	0.5
4	59°C	1
5	Repeat step 3-4 39x	
6	98°C	10
7	4°C	Indefinite
**Two-step ddPCR**		
Step	*Temp*	*Minutes*
1	95°C	5
2	95°C	0.5
3	60°C	1
4	Repeat step 3-4 39x	
5	4°C	5
6	90°C	10
7	4°C	Indefinite

Supplementary Table 6. Primary antibodies and probes

**Antibody or probe**	**Supplier**	**Dilution**
DAPI (nuclear stain)	Sigma-Aldrich	2 µg/mL
Mouse anti-Pericentrin	Abcam	1:1000
Rabbit anti-Arl13b	ProteinTech	1:1000
Rabbit anti-recoverin	Millipore	1:500
Mouse anti-cone arrestin clone 7G6	kind gift of Wolfgang Baehr	1:100

Supplementary Table 7. In vivo doses and corresponding concentration in the vitreous humor

**Cynomolgus Monkey (Vitreal Volume 1.53 mL)**	
IVT dose	Vitreal Conc.
60 µg	6.22 µM
100 µg	10.37 µM
900 µg	93.37 µM
**Dutch Belted Rabbit (Vitreal Volume 1.12 mL)**	
IVT dose	Vitreal Conc.
100 µg	14.17 µM

Supplementary Table 8. List of differentially expressed gene in LCA10 retinal organoids following 1 μM QR-110 treatment.

Gene ID	Gene Length	Mean Untreated	Mean QR-110	log2Ratio (QR-110 / Untr)	Regulation QR-110 / Untr	Probability	Symbol
100526830	2218	2.59	0.01	-8.017	Down	0.802	SLX1A-SULT1A3
642799	1088	19.585	5.3	-1.886	Down	0.860	NPIPA2
8293	1935	13.675	3.835	-1.834	Down	0.844	SERF1A
4741	3271	46.13	13.47	-1.776	Down	0.870	NEFM
4747	3854	45.55	16.175	-1.494	Down	0.847	NEFL
100874097	443	13.025	4.96	-1.393	Down	0.803	UBE2Q1-AS1
2785	962	33.425	15.885	-1.073	Down	0.802	GNG3
2878	1805	77.88	162.59	1.062	Up	0.814	GPX3
1298	2831	12.375	26.565	1.102	Up	0.801	COL9A2
59	1460.21	23.015	49.85	1.115	Up	0.815	ACTA2
344658	2399.03	13.72	30.41	1.148	Up	0.811	SAMD7
9284	1102	13.385	30.09	1.169	Up	0.813	NPIPA1
1278	5411	65.91	150.74	1.193	Up	0.829	COL1A2
6029	299	23.635	54.24	1.198	Up	0.824	RN7SL1
1281	5490	76.075	175.23	1.204	Up	0.831	COL3A1
3490	1137	8.93	20.81	1.221	Up	0.808	IGFBP7
2719	2329	20.165	50.75	1.332	Up	0.835	GPC3
1277	5927	92.375	233.665	1.339	Up	0.843	COL1A1
2537	837.62	11.09	29.15	1.394	Up	0.833	IFI6
3856	1812.95	5.88	16.695	1.506	Up	0.827	KRT8
10457	2757.64	6.09	17.37	1.512	Up	0.828	GPNMB
2697	3169	13.085	39.775	1.604	Up	0.858	GJA1
6085	102	6.905	23.705	1.779	Up	0.860	RNY3
7980	2444	8.6	31.79	1.886	Up	0.870	TFPI2
9076	3452	5.11	19.105	1.903	Up	0.860	CLDN1
6490	2141.53	7.925	32.335	2.029	Up	0.880	PMEL
245912	189	1.955	8.475	2.116	Up	0.833	DEFB109P1
3294	1451	2.645	11.47	2.117	Up	0.853	HSD17B2
729330	1782	10.15	44.435	2.130	Up	0.887	OC90
4308	4867.98	2.945	13.005	2.143	Up	0.860	TRPM1
646960	2158	1.585	7.715	2.283	Up	0.831	PRSS56
2792	720	1.84	9.25	2.330	Up	0.851	GNGT1
1306	5422	1.605	8.21	2.355	Up	0.843	COL15A1
314	2681	2.27	12.03	2.406	Up	0.869	AOC2
101929127	430.83	1.055	5.925	2.490	Up	0.816	PGM5P3-AS1
7306	2876	2.285	13.405	2.553	Up	0.877	TYRP1
1634	4309.39	2.02	14.665	2.860	Up	0.894	DCN

Gene ID	Gene Length	Mean Untreated	Mean QR-110	log2Ratio (QR-110 / Untr)	Regula-tion QR-110 / Untr	Probability	Symbol
7299	2082	0.755	5.49	2.862	Up	0.826	TYR
22915	4973	0.69	5.185	2.910	Up	0.820	MMRN1
7276	938	1.005	8.825	3.134	Up	0.875	TTR
642778	1098	0.025	6.505	8.023	Up	0.931	NPIPA3
26829	119	0.01	3.115	8.283	Up	0.837	RNU5E-1
6705	671	0.01	3.44	8.426	Up	0.855	SPRR2F
101929829	1666	0.01	3.45	8.430	Up	0.855	LOC101929829

Supplementary Methods

Sequencing

CEP290 c.5668G>T (p.Gly1890X) mutant transcripts were amplified using the AmpliTaq Gold® 360 DNA polymerase kit (Thermo Scientific) with standard cycling conditions and ran on Bioanalyzer (Agilent 2100) to check the size on a DNA1000 chip (Agilent). Primer sequences are summarized in **Supplementary Table 4**. Results were analyzed using Agilent2100 expert software. The PCR product was directly purified using the NucleoSpin® Gel and PCR-clean up (Macherey-Nagel) according to manufacturer's protocol and sent to BaseClear (Leiden, The Netherlands) for Sanger sequencing.

PBMC viability and stimulation assay

Buffy coats of 5 consenting healthy human blood donors were collected (Sanquin Blood Bank, Leiden, the Netherlands) and PBMC were isolated using Ficoll Paque (GE Healthcare) density gradient centrifugation according to the manufacturer's instructions within 24 hours after buffycoat extraction. PBMC were cryopreserved in medium containing 10% DMSO and stored at -150˚C until use. Cryopreserved PBMC were thawed and viability was assessed by trypan blue exclusion method. Assay was performed only if the PBMC viability was >95%. PBMC were cultured in 96-well microtiter plates ($1.0*10^5$ per well) for 24 hours in a humidified incubator at 37˚C along with lead candidates 1, 2 and 3 (10nM, 100nM or 1µM each), with positive controls Poly(I:C) (tlr-picw, Invivogen) (1µg/ml), CpG (tlrl-bw006, Invivogen) (10µg/ml), LPS (Tlrl-3pelps, Invivogen) (100ng/ml) and R848 (Invivogen) (1µM), or with saline. All conditions were assayed in triplicate. Cell culture supernatants were collected and stored at -80˚C for cytokine measurement at a later moment. Cytokine levels were measured using the Bio-Plex Pro™ Human Cytokine 17-plex Assay (Bio-Rad) on a Luminex MAGPIX platform (Luminex Corporation) according to the manufacturer's recommendations. After removal of the supernatant, viability of the PBMC was assessed on the remaining cells of the PBMC stimulation assay using the CellTiter-Blue reagent (Promega) according to the manufacturer's instructions. Each measurement was normalized against its corresponding saline control. Repeated measures One-way ANOVA with Dunnett's test for multiple comparisons (compared to saline) was performed on the fold change

values, calculated by normalization against the geometric mean of their corresponding triplicate saline control.

Transcriptome analysis

Samples were sequenced using BGISEQ-500RS (BGI Europe, Denmark). On an average 24 million clean reads per sample were generated and mapped to GRCh38 human reference genome using HISAT/Bowtie2. Raw data and processed files are available at gene expression omnibus (GEO) database repository with the accession number GSE116797.
